# The 2‐Arsaethynolate Anion: Synthesis and Reactivity Towards Heteroallenes

**DOI:** 10.1002/anie.201602310

**Published:** 2016-04-20

**Authors:** Alexander Hinz, Jose M. Goicoechea

**Affiliations:** ^1^ Department of ChemistryUniversity of Oxford, Chemistry Research Laboratory12 Mansfield RoadOX1 3TAOxfordUK

**Keywords:** arsenic, cluster compounds, cycloadditions, reaction mechanisms, structure elucidation

## Abstract

The synthesis and isolation of the 2‐arsaethynolate anion, AsCO^−^, and its subsequent reactivity towards heteroallenes is reported. Reactions with ketenes and carbodiimides afford four‐membered anionic heterocycles in formal [2+2] cycloaddition reactions. By contrast, reaction with an isocyanate yielded a 1,4,2‐diazaarsolidine‐3,5‐dionide anion and the unprecedented cluster anions As_10_^2−^ and As_12_^4−^. These preliminary reactivity studies hint at the enormous potential synthetic utility of this novel anion, which may be employed as an arsenide (As^−^) source.

The 2‐phosphaethynolate anion, PCO^−^ (a heavier analogue of cyanate, NCO^−^), was first reported by Becker et al. in 1992.^1^ Following this seminal report, several additional synthetic routes to this species have been documented, thus allowing for the bulk preparation of alkali metal salts of the anion in high yields.^2^, ^3^, ^4^, ^5^, ^6^ In the last five years, this species has gone from being a chemical curiosity to a versatile chemical precursor for a range of phosphorus‐containing compounds, including heterocycles,^5^, ^6^, ^7^, ^8^, ^9^, ^10^ low‐valent compounds,^10^, ^11^ and novel phosphines.^12^, ^13^, ^14^ The coordination chemistry of the anion to d‐block and f‐block metals has also been explored.^15^, ^16^, ^17^, ^18^ The synthetic scope offered by PCO^−^, which upon spontaneous or photolytic decarbonylation can be employed as a source of monoanionic phosphorus (P^−^),^9^, ^11^ makes the isolation of related chemical compounds highly desirable.

Heavier group 15 analogues of PCO^−^ are unknown. Early attempts by Hübler and Becker to generate the 2‐arsaethynolate anion, AsCO^−^, yielded Li_3_As_7_.^19^ Theoretical studies have nevertheless indicated the stability of the heavier members of the homologous ECO^−^ series (E=As, Sb, Bi).^20^ These studies prompted us to address the question posed by Lu, Schaefer, and co‐workers: “These successes in transitioning from NCO^−^ to PCO^−^ raise the obvious question: can heavier congeners of PCO^−^ be made?”. Herein we report our efforts to reinvestigate the synthesis of the AsCO^−^, which until now has only been isolated in matrix isolation experiments.^21^

AsCO^−^ can be prepared by following a modified version of the synthetic procedure reported by Grützmacher for PCO^−^ (Scheme 1).^6^ Reaction of elemental sodium and arsenic in a 3:1 ratio in dimethoxyethane (DME) with catalytic amounts of naphthalene affords a black suspension containing Na_3_As. Critically, this step requires prolonged reaction times and stirring for up to three days at 70°C. Protonation using a sterically encumbered alcohol (*t*BuOH) followed by carbonylation using diethylcarbonate affords a solution of AsCO^−^ (**1**). To aid solubility and facilitate the crystallographic characterization of the anion 1,4,7,10,13,16‐hexaoxacyclooctadecane (18‐crown‐6) can be added to the mixture in the final stage. The product can be precipitated out of solution by addition of 1,4‐dioxane and isolated as a yellow powder. This compound can subsequently be recrystallized from a number of different solvent/anti‐solvent mixtures, affording a colorless crystalline material in moderate to high yields (approx. 60 %).

**Scheme 1:**
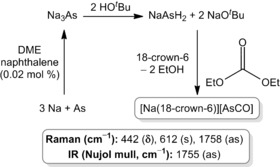
Synthesis and vibrational data for [Na(18‐crown‐6)][**1**].

The structure of the anion was determined by single‐crystal X‐ray diffraction. Three different solvates were isolated by varying the solvent of crystallization and/or reaction conditions: [Na(18‐crown‐6)][**1**]⋅(C_4_H_8_O_2_)_0.5_, [Na(18‐crown‐6)(NC_5_H_5_)_2_][**1**], and [Na(18‐crown‐6)_1.5_][**1**]. Of the three, the latter two display positional disorder of the anion, and are therefore not suitable for the discussion of bond metric data (the structures are provided in the Supporting Information). [Na(18‐crown‐6)][**1**]⋅(C_4_H_8_O_2_)_0.5_ contains a single anion in the asymmetric unit accompanied by [Na(18‐crown‐6)]^+^ and half a molecule of 1,4‐dioxane (Figure 1). The structure clearly shows a linear anion with an As1‐C1‐O1 angle of 176.6(2)°. The As−C and C−O bond lengths are 1.707(3) and 1.197(3) Å, respectively. The As−C distance lies between the values expected for a double (1.81 Å) and triple bond (1.66 Å),^22^ and is somewhat shorter than previously predicted computationally for **1** (1.740 Å).^20^ This difference may be due to the relatively short contact arising between Na1 and O1 [2.290(2) Å], which would have the effect of increasing the triple‐bond character of the As−C bond. However, it is also worth noting that the structure of **1** was computed in the gas phase, and that therefore no solvation effects and/or Coloumbic interactions were taken into consideration.

**Figure 1:**
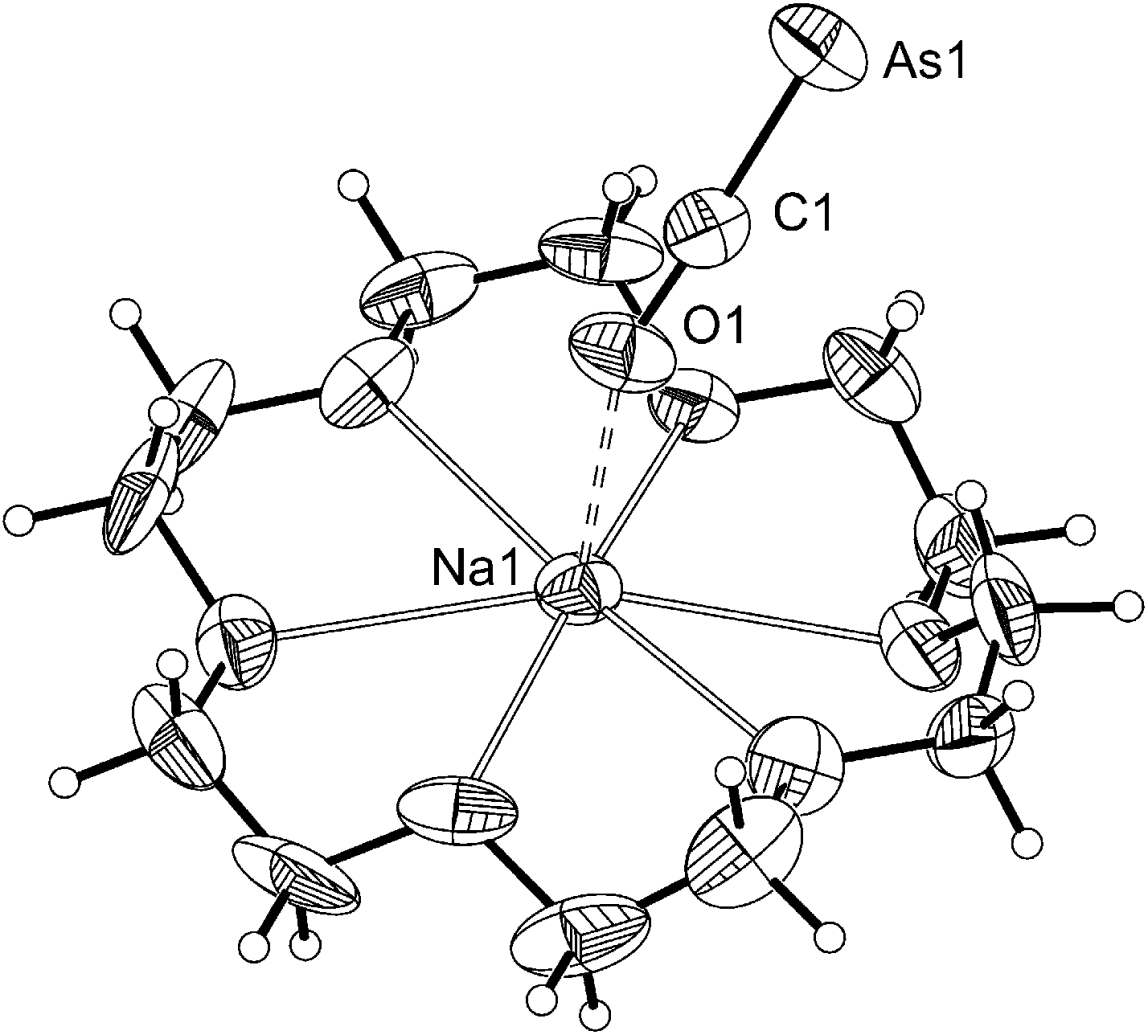
Thermal ellipsoid plot of the atoms in the asymmetric unit of [Na(18‐crown‐6)][**1**]⋅(C_4_H_8_O_2_)_0.5_. Anisotropic thermal displacement ellipsoids are pictured at the 50 % probability level at 150K. Solvent of crystallization and disordered components of the 18‐crown‐6 moiety have been removed for clarity. Hydrogen positions were assigned idealized coordinates and are pictured as spheres of arbitrary radius. Selected bond distances (Å) and angles (°): As1–C1, 1.707(3); C1–O1, 1.197(3); O1⋅⋅⋅Na1, 2.290(2); As1‐C1‐O1, 176.6(2).

As for its lighter homologues, two main resonance forms can be proposed for **1**, either with localization of the negative charge on the oxygen atom and a formal As≡C triple bond [**1_a_** as depicted in Scheme 2], or a structure with a formal As=C bond and a negative charge on arsenic [**1_b_**]. Natural bond orbital (NBO) calculations revealed Wiberg bond indices (WBI) of 2.14 and 1.70 for the As−C and C−O bonds, respectively, and atomic charges of −0.50 and −0.60 for the arsenic and oxygen atoms, respectively.^20^ These data are similar to those obtained for PCO^−^, and reveal that both resonance forms contribute significantly to bonding, thus allowing for an effective delocalization of negative charge along the anion. A natural resonance theory (NRT) analysis weighs the relative contributions of the ethynolate (As≡C‐O^−^) and ketenide (^−^As=C=O) resonance forms as 35 and 48 %, respectively.

**Scheme 2:**
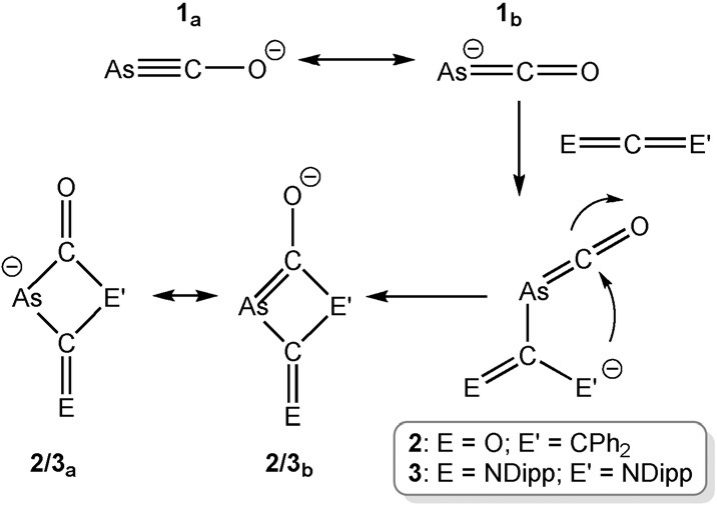
Synthesis of **2** and **3** from **1** and diphenylketene and bis(2,6‐diisopropylphenyl)carbodiimide, respectively.

The vibrational spectra obtained for **1** are consistent with values reported from matrix isolation studies.^21^ The Raman spectrum of [Na(18‐crown‐6)][**1**]⋅(C_4_H_8_O_2_)_0.5_ exhibits three bands at 444, 630, and 1746 cm^−1^, which arise from the corresponding bending, symmetric, and asymmetric stretching modes (cf. 444, 609, and 1756 cm^−1^ as reported by Zhang et al.). Interestingly, we were able to observe variations in the data depending on whether or not the anion interacts with the charge‐balancing cation, thus Raman bands for [Na(18‐crown‐6)_1.5_][**1**], where the anion shows no close contacts with Na^+^, were observed at 442, 612, and 1758 cm^−1^, and are an even better match with previously reported data.

The ^13^C NMR spectrum of [Na(18‐crown‐6)][**1**]⋅(C_4_H_8_O_2_)_0.5_ in [D_8_]THF exhibits a singlet at *δ*=179.4 ppm arising from **1**, in addition to a singlet at *δ*=71.1 ppm arising from the crown ether. A resonance at *δ*=3.55 ppm in the ^1^H NMR spectrum also arises from 18‐crown‐6.

Having developed a large‐scale synthesis for AsCO^−^ we were prompted to explore its reactivity in an effort to establish comparisons with the chemistry of its lighter homologues NCO^−^ and PCO^−^. Our initial studies show that AsCO^−^ is highly susceptible to oxidation, readily affording As_7_^3−^ (as observed in early experiments by Becker and co‐workers) or elemental arsenic when exposed to trace amounts of air or mild oxidants. This heightened reactivity makes it much less convenient to work with than its phosphorus‐containing analogue. Of the reactions we have explored thus far, the most successful involve the reaction of AsCO^−^ with unsaturated organic substrates such as heteroallenes. Thus, reaction of AsCO^−^ with selected ketenes (O=C=CPh_2_) and carbodiimides (DippN=C=NDipp; Dipp=2,6‐diisopropylphenyl) afford the anionic [2+2] cycloaddition products As[C(O)]_2_CPh_2_^−^ (**2**) and AsC(O)(CNDipp)NDipp^−^ (**3**), respectively (Scheme 2). The reactions were monitored by ^13^C NMR spectroscopy and were found to proceed to completion by the time the NMR data were collected. Analogous reactivity has been reported by our research group for the lighter PCO^−^ analogue, which exhibits similar shifts for the resonances of the quartenary carbon atoms in the small heterocycles [**2**: 106.8 (*C*CO), 230.2 (*C*=*O*), cf. P[C(O)]_2_CPh_2_^−^: 95.7, 225.1 ppm; **3**: 178.6 (*N*‐*C*=*N*), 195.7 (*C*=*O*), cf. PC(O)(CNDipp)NDipp^−^: 178.2, 196.6ppm].^5^

As[C(O)]_2_CPh_2_^−^ (**2**) and AsC(O)(CNDipp)NDipp^−^ anions (**3**) were structurally characterized as [Na(18‐crown‐6)]^+^ salts (Figure 2). Both structures reveal a four‐membered ring system with acute C1‐As1‐C2 bond angles [71.12(14) and 67.43(9)°, for **2** and **3**, respectively] which are similar to those recorded for the analogous phosphorus‐containing compounds. The As−C bond lengths [**2**: 1.925(4), 1.941(4) Å; **3**: 1.961(2) and 1.976(2) Å], are logically longer than the corresponding P−C bonds in the lighter homologues, and notably shorter for **2** when compared to **3**. This difference is the result of negative‐charge delocalization over the two carbonyl moieties, and confers greater double‐bond character to the As−C bonds. In contrast the backbone imino functionality (NDipp) present in **3** has a pair of electrons in a π‐type orbital orthogonal to the four‐membered ring which can also be delocalized into the adjacent carbonyl groups, thus lengthening the As−C bonds. Nucleus independent chemical shift (NICS) calculations show anti‐aromatic character for **2** and non‐aromatic character for **3** [NICS(0)=+7.8 and *δ*=−2.4 ppm for **2** and **3**, respectively]. The C1–O1/C2–O2 and C1–O1/C2–N2 bond lengths in **2** [1.227(5)/1.218(5) Å] and **3** [1.217(3)/1.265(3) Å] are characteristic of double bonds. Structurally authenticated examples of such four membered ring systems containing arsenic are rare and include As_4_^2−^, Cp*C(NR)_2_As[W(CO)_5_]_2_ (R=isopropyl or cyclohexyl, Cp*=pentamethylcyclopentadienyl) and EAs(NAr)_2_ (E=N, P, As; Ar=2,6‐dimesitylphenyl).^23^, ^24^, ^25^

**Figure 2:**
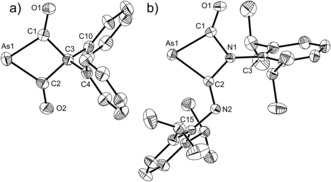
Thermal ellipsoid plots of **2** (left) and **3** (right). Anisotropic thermal displacement ellipsoids are pictured at the 50 % probability level at 150K. [Na(18‐crown‐6)]^+^ and hydrogen atoms have been removed for clarity. Selected bond distances (Å) and angles (°) **2**: As1–C1, 1.925(4); As1–C2, 1.941(4); C1–C3, 1.552(4); C2–C3, 1.558(5); C1–O1, 1.227(5); C2–O2, 1.218(5); C3–C4, 1.527(5); C3–C10, 1.510(5); C1‐As1‐C2, 71.12(14); As1‐C1‐C3, 98.5(2); C1‐C3‐C2, 92.6(3); As1‐C2‐C3, 97.6(2). O1 displays a short interatomic contact to the sodium cation of 2.285(3) Å. **3**: As1–C1, 1.961(2); As1–C2, 1.976(2); C1–N1, 1.414(3); N1–C2, 1.411(3); C1–O1, 1.217(3); N1–C3, 1.441(3); C2–N2, 1.265(3); N2–C15, 1.421(3); C1‐As1‐C2, 67.43(9); As1‐C1‐N1, 95.91(14); C1‐N1‐C2, 101.32(17); As1‐C2‐N1, 95.34(14). O1 displays a short interatomic contact to the sodium cation of 2.269(2) Å.

By contrast with the aforementioned studies, reactions between **1** and isocyanates (O=C=NDipp) proceed less cleanly and their outcome is strongly dependent on the stoichiometric loading of reagents. From such reaction mixtures we were able to isolate the five‐membered cyclic compound As[C(O)]_2_[NDipp]_2_^−^ (**4**; Figure 3) which can be obtained as a compositionally pure species by fractional crystallization. Analogous phosphorus‐containing ring systems have previously been reported from similar reactions between PCO^−^ and O=C=NDipp.^10^ **4** can be considered as the result of the cyclization of one molecule of **1** with two equivalents of isocyanate accompanied by loss of carbon monoxide (Scheme 3). ^13^C NMR spectroscopic studies on these reaction mixtures revealed that **4** is the only product formed incorporating organic functionalities. Decarbonylation occurs readily for reactions involving **1**, and when a low stoichiometric loading of O=C=NDipp was employed, a red crystalline solid could also be obtained and consisted of a solid solution of three different arsenic clusters As_10_^2−^ (pseudo *D*_2*h*_) and As_12_^4−^ (the latter occurring as two different isomers: *C*_2*h*_ and *D*_4*h*_). These cluster anions can be thought of as intermediates in the oxidative decarbonylation of AsCO^−^ and, as with the well‐known As_7_^3−^ cluster, and represent snapshots of a process which ultimately affords elemental arsenic and carbon monoxide.

**Figure 3:**
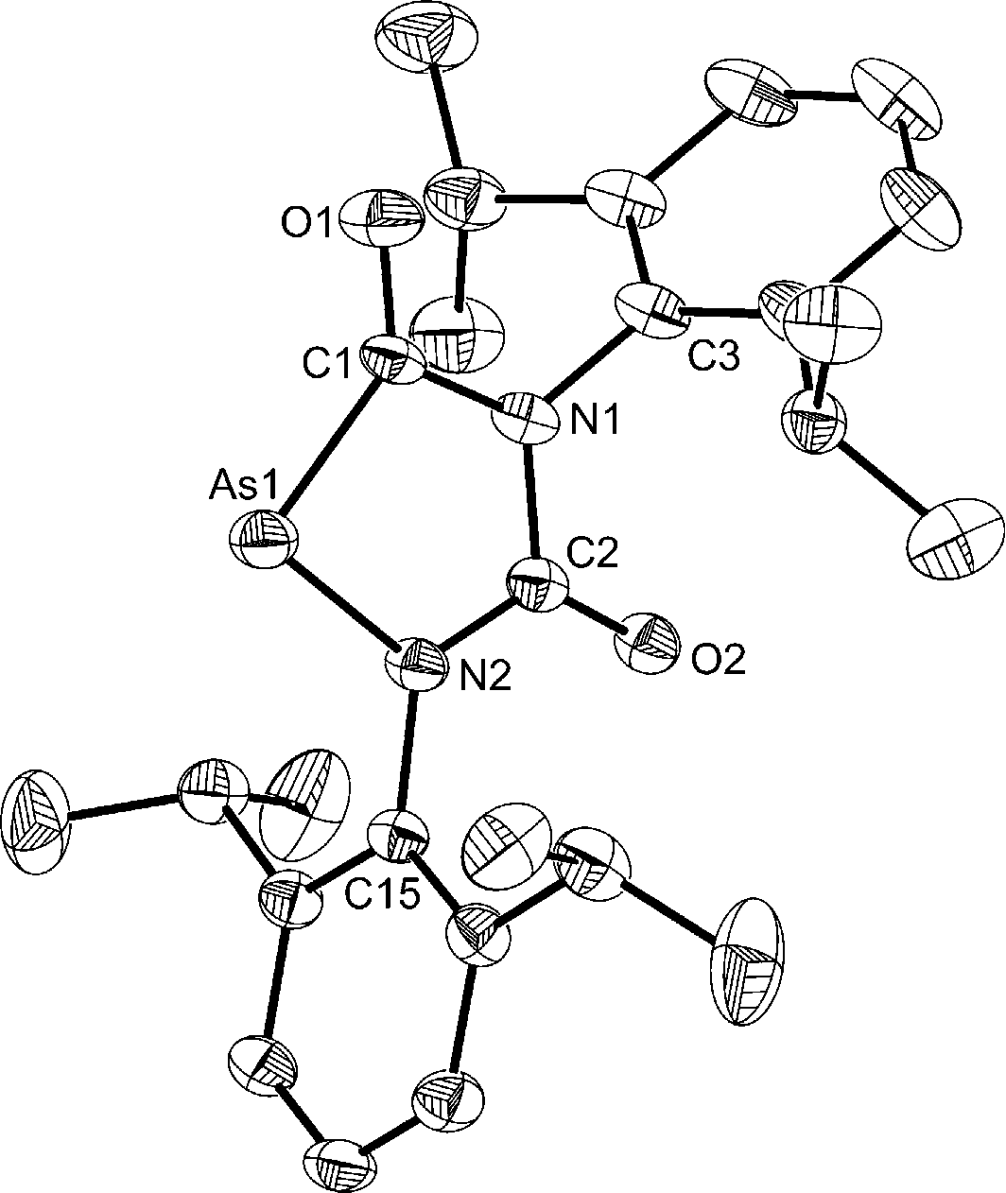
Thermal ellipsoid plot of the atoms in the asymmetric unit of [Na(18‐crown‐6)][**4**]. Anisotropic thermal displacement ellipsoids are pictured at the 50 % probability level at 150K. [Na(18‐crown‐6)]^+^ and hydrogen atoms have been removed for clarity. Selected bond distances (Å) and angles (°): As1–C1, 1.898(2); C1–O1, 1.200(3); C1–N1, 1.462(3); N1–C2, 1.404(2); N1–C3, 1.430(3); C2–O2, 1.238(3); C2–N2, 1.341(3); N2–C15, 1.432(2); N2–As1, 1.914(2); C1‐As1‐N2, 85.21(8); As1‐C1‐N1, 108.06(12); C1‐N1‐C2, 119.18(16); N1‐C2‐N2, 110.23(17); C2‐N2‐As1, 117.30(13). O2 displays a short interatomic contact to the sodium cation of 2.362(2) Å.

**Scheme 3:**
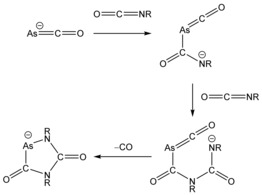
Synthesis of **4** from **1** and bis(2,6‐diisopropylphenyl)isocyanate. R=2,6‐Diisopropylphenyl.

The single‐crystal X‐ray structure of **4** reveals a single five‐membered ring system in the asymmetric unit accompanied by a charge‐balancing [Na(18‐crown‐6)]^+^ cation. It crystallizes in the space group *P*2_1_/*n* and is isomorphous with its phosphorus‐containing analogue [K(18‐crown‐6)][P[C(O)]_2_(NDipp)_2_].^10^ As expected, the structural differences between both anions are largely insignificant. The N‐As‐C bond angle is 85.21(8)°, which is slightly more acute than the corresponding angle in the phosphorus‐containing species [90.7(1)°], and is in line with the longer As−C and As−N bonds [1.898(2) and 1.914(2) Å, respectively]. The C2–O2 bond length is slightly longer than C1–O1 [1.238(3) vs. 1.200(3) Å], and can be explained by the fact that O2 exhibits a close contact in the lattice with the sodium cation [2.362(2) Å]. The ^13^C NMR data of **4** compare well to the known phosphorus analogue [**4** in [D_8_]‐THF: 179.4 (N*C*(N)O), 204.5 (As*C*O); [P[C(O)]_2_(NDipp)_2_]^−^ in [D_3_]‐MeCN: *δ*=153.8, 195.8 ppm].^10^

A red crystalline solid could be obtained as a side product from reactions involving isocyanates and AsCO^−^. A single‐crystal X‐ray diffraction analysis of this sample revealed three [Na(18‐crown‐6)]^+^ cations in the asymmetric unit. The packing of such cations leaves space for a single cluster species. Close inspection of the heavily disordered cluster site revealed dianionic As_10_^2−^, and a tetraanionic As_12_^4−^ species, both of which occupy the site in a 1:1 ratio (hence giving rise to a net 3− charge). The latter species exhibits two different topologies (Figure 4 b and 4c) with *C*_2*h*_ and *D*_4*h*_ symmetry (with relative occupancies of 36 and 14 %, respectively). All three cluster anions are electron‐precise species in which the arsenic atoms that are bonded to three adjacent vertices are neutral, and those that are only bonded to two adjacent atoms carry formal negative charges.

**Figure 4:**
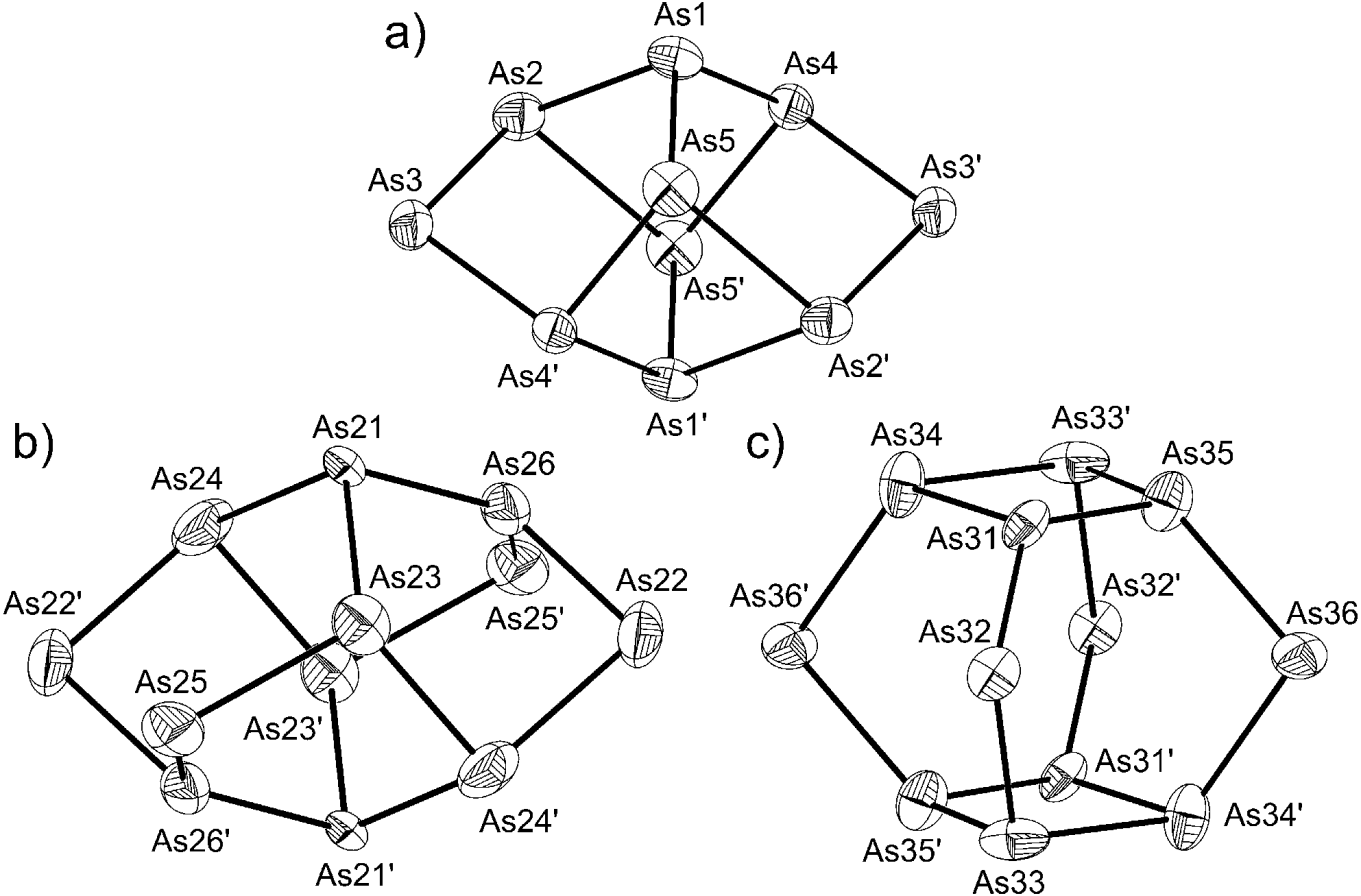
The three different cluster anions present in [Na(18‐crown‐6)]_3_[As_10_]_0.5_[As_12_]_0.5_⋅4 THF. a) As_10_^2−^ which is present in the lattice with an occupancy of 50 %. b) As_12_^4−^ (*C*_2*h*_), and c) As_12_^4−^ (*D*_4*h*_) which have occupancies of 35 % and 15 %, respectively. Anisotropic thermal displacement ellipsoids are pictured at the 50 % probability level at 150 K.

The As_10_^2−^ geometry is related to that of the well‐known As_7_^3−^. The latter species adopts a norbornadiene‐like geometry when coordinated to metal atoms in clusters such as [As_7_Cr(CO)_3_]^3−^.^26^, ^27^ As_10_^2−^ can be thought of as two such norbornadiene‐like clusters fused through the four atoms through which they characteristically would bond to a metal center. As such the cluster exhibits a distorted *D*_2*h*_ geometry. The optimized geometry obtained by theoretical calculations at the density functional level of theory (DFT) are consistent with such a geometry. The As_12_^4−^ cluster adopts two different geometries, a low‐symmetry *C*_2*h*_ isomer and a higher symmetry, but thermodynamically less stable, *D*_4*h*_ geometry. DFT calculations showed the latter species to be 73 kJ mol^−1^ higher in energy, and may account for its lower abundance in the crystal lattice. It is interesting to note that the combination of As_10_^2−^ and As_12_^4−^ is energetically not favorable compared to the known As_11_^3−^ cluster anion by 437 kJ mol^−1^ per ion pair (see Table S5 in the Supporting Information).^28^ The structures exhibited by these anions are without precedent for the elements of group 15 and indicate that, upon oxidation, AsCO^−^ may be employed as a source of metastable homoatomic arsenic clusters. As mentioned above, early studies by Becker and co‐workers aimed at isolating AsCO^−^ resulted in the isolation of the Zintl ion As_7_^3−^, a related electron‐precise cluster.

In summary, we have developed a synthetic route to AsCO^−^ by carbonylation of NaAsH_2_. This method afforded AsCO^−^ (**1**) in good yields, thus allowing subsequent reactivity studies which have demonstrated that cyclizations between the As−C bond in **1** and unsaturated substrates are possible and yield novel heterocyclic species. Studies are currently underway further exploring the reactivity of **1** towards a library of compounds.

## Supporting information

As a service to our authors and readers, this journal provides supporting information supplied by the authors. Such materials are peer reviewed and may be re‐organized for online delivery, but are not copy‐edited or typeset. Technical support issues arising from supporting information (other than missing files) should be addressed to the authors.

Supplementary
