# Supplementary material for: The 2‐Arsaethynolate Anion: Synthesis and Reactivity Towards Heteroallenes
Source: Angew Chem Int Ed Engl. 2016 Apr 20;55(30):8536–41. doi: 10.1002/anie.201602310 (PMC5074235; doi:10.1002/anie.201602310)
Supplement: Supplementary file 1 — Supplementary [file ANIE-55-8536-s001.pdf]

Angewandte Chemie
Eine Zeitschrift der Gesellschaft Deutscher Chemiker

Supporting Information

The 2-Arsaethynolate Anion: Synthesis and Reactivity Towards Heteroallenes

*Alexander Hinz and Jose M. Goicoechea**

anie_201602310_sm_miscellaneous_information.pdf

1. Experimental section....2
1.1 Synthesis of [Na(18-crown-6)][AsCO] ([Na(18-crown-6)][**1**])....4
1.2 Synthesis of [Na(18-crown-6)][As[C(O)]$_2$CPh$_2$] ([Na(18-crown-6)][**2**])....8
1.3 Synthesis of [Na(18-crown-6)][AsC(O)(CNDipp)NDipp] ([Na(18-crown-6)][**3**])....10
1.4 Synthesis of [Na(18-crown-6)][As[C(O)]$_2$(NDipp)$_2$] ([Na(18-crown-6)][**4**]) and [Na(18-crown-6)]$_6$[As$_{10}$][As$_{12}$]....12
2. Crystallographic data....15
3. Computational data....20
3.1 Optimized Geometries....21
3.1.1 $AsCO^{-}$....21
3.1.2 [Na(18-crown-6)(dioxane)][AsCO]....21
3.1.3 $[As[C(O)]_2CPh_2]^{-}$ (**2**)....22
3.1.4 $[AsC(O)(CNDipp)NDipp]^{-}$ (**3**)....23
3.1.5 As_{10}^{2-}....25
3.1.6 As_{12}^{4-}....26
3.1.7 As_{12}^{4-}....26
3.1.8 As_{11}^{3-}....27
3.1.9 $[As[C(O)]_2(NDipp)_2]^{-}$ (**4**)....27
4. References....29

1. Experimental section

General synthetic methods. All reactions and product manipulations were carried out under an inert atmosphere of argon or dinitrogen using standard Schlenk-line or glovebox techniques (MBraun UNIlab glovebox maintained at < 0.1 ppm H_2O and < 0.1 ppm O_2). Diphenylketene and *N*,*N*'-bis-(2,6-diisopropylphenyl)-carbodiimide were prepared according to known procedures.[1]

Sodium (99.8%, Acros) and arsenic (99%, Alfa Aesar) were purchased from commercial sources. Diethylcarbonate (99%, Aldrich) and 2,6-diisopropylphenyl-isocyanate (98%, Aldrich) were used as received. 18-crown-6 (1,4,7,10,13,16-hexaoxacyclooctadecane; 99%, Alfa Aesar) was purified by distillation prior to use. Naphthalene was purified by sublimation prior to use. *Tert*-butanol (99%, Alfa Aesar) was dried over sodium. Hexane (hex; Sigma-Aldrich, HPLC grade) and dimethylformamide (DMF; Rathburn, 99.9%) were purified using an MBraun SPS-800 solvent system. Tetrahydrofuran (THF; Sigma-Aldrich, HPLC grade), dimethoxyethane (Alfa Aesar, 99%), and 1,4-dioxane (Fisher, p.a) were distilled over sodium metal/benzophenone. Pyridine (py; Alfa Aesar, 99%) was dried over CaH_2 and distilled prior to use. [D_8]-THF (Sigma–Aldrich, 99.5%) and [D_5]-pyridine were dried over CaH_2 and vacuum distilled before use. All dry solvents were stored under argon in gas-tight ampoules. Additionally hexane and THF were stored over activated 3 Å molecular sieves.

Single crystal X-ray structure determination: Single-crystal X-ray diffraction data were collected using either an Oxford Diffraction Supernova dual-source diffractometer equipped with a 135 mm Atlas CCD area detector. Crystals were selected under Paratone-N oil, mounted on micromount loops and quench-cooled using an Oxford Cryosystems open flow N_2 cooling device.[2] Data were collected at 150 K using mirror monochromated Cu K_α

radiation (λ = 1.5418 Å; Oxford Diffraction Supernova). Data were processed using the CrysAlisPro package, including unit cell parameter refinement and inter–frame scaling (which was carried out using SCALE3 ABSPACK within CrysAlisPro).[3] Equivalent reflections were merged and diffraction patterns processed with the CrysAlisPro suite. Structures were subsequently solved using direct methods and refined on F^2 using the SHELXL 2013 package.[4]

Additional characterization techniques: ^{1}H, ^{13}C and ^{31}P NMR spectra were acquired at 500.0, 125.7 and 202.4 MHz, respectively, on a Varian Unity Plus 500 NMR spectrometer at 298 K if not stated otherwise. ^{1}H and ^{13}C NMR spectra were referenced to the most downfield solvent resonance (^{1}H NMR [D_8]-THF: δ = 3.58 ppm; ^{13}C NMR [D_8]-THF: δ = 67.2 ppm).[5] ^{31}P spectra were externally referenced to an 85% solution of H_3PO_4 in H_2O (δ = 0 ppm).

Elemental analyses were carried out by Elemental Microanalyses Ltd. (Devon, U.K.). Samples (approx. 10 mg) were submitted in sealed Pyrex ampoules.

1.1 Synthesis of [Na(18-crown-6)][AsCO] ([Na(18-crown-6)][1])

Sodium (2.300 g, 100 mmol; in small pieces), arsenic (2.498 g, 33.3 mmol; powder), and naphthalene (200 mg, 1.56 mmol) were combined in a Schlenk flask. Then, 100 ml of dimethoxyethane (DME) were added, immediately forming a green solution. The mixture was stirred with a glass-covered stirring bar at 70 °C for three days, forming a dark greenish solution and black microcrystalline precipitate. To the suspension, *tert*-butanol (4.940 g, 66.6 mmol) was added via syringe. The suspension was stirred for six hours. When all solid had dissolved the solution had turned greenish-yellow. In another flask, 18-crown-6 (26.410 g, 100 mmol) was dissolved in 20 ml of DME and the colourless solution was added to the reaction mixture, which caused the reaction mixture to become turbid. After stirring for 30 minutes at ambient temperature, diethylcarbonate (3.940 g, 33.3 mmol) was added via syringe and the suspension was stirred overnight. The initially greenish-yellow suspension turned orange-yellow during this process. All volatiles were removed *in vacuo* at ambient temperature. To the pale yellowish residue 150 ml of THF were added, resulting in the formation of a dark yellow suspension. After vigorous stirring for 1 hour, the mixture was left to settle overnight. The mixture was filtered over a Celite-padded sinter, affording a clear yellow solution, which was then concentrated to approximate half the volume. Then an approximate four-fold amount of 1,4-dioxane was added to precipitate the crude product. The solution was filtered off and the yellow solid was dried under a dynamic vacuum, affording 10.727 g of crude product. The product was redissolved in a minimum amount of THF (approximately 5 ml/g) and layered with hexane afterwards. After standing undisturbed overnight, large colourless crystals of [Na(18-crown-6)(dioxane)$_{0.5}$][AsCO] formed. The supernatant was removed via cannula and the crystals were dried for *in vacuo* at ambient temperature for 8 hours, affording [Na(18-crown-6)][AsCO] as colourless substance (7.838 g, 20.1 mmol, 60%).

Recrystallization of the crude product by layering a pyridine solution with hexane afforded [Na(18-crown-6)(pyridine)$_2$][AsCO], which can also be desolvated by prolonged drying under vacuum.

[Na(18-crown-6)$_{1.5}$][**1**]

CHN for [Na(18-crown-6)$_{1.5}$][**1**] found % (calc. %): C 44.17 (43.68), H 7.08 (6.95).

^{1}H NMR ([D$_5$]-pyridine): 3.52 (s, crown).

^{13}C{^{1}H} NMR ([D$_5$]-pyridine): 181.3 (s, As*C*O), 70.8 (s, crown).

^{1}H NMR ([D$_8$]-THF): 3.61 (s, crown).

^{13}C{^{1}H} NMR ([D$_8$]-THF): 179.4 (s, As*C*O), 71.1 (s, crown).

Raman (cm^{-1}): 442 (9, δ), 612 (100, ν_s), 1758 (15, ν_{as}).

IR (Nujol mull, cm^{-1}): 1755 (w, ν_{as}).

[Na(18-crown-6)][**1**]·($C_4H_8O_2$)$_{0.5}$

CHN for [Na(18-crown-6)$_{1.5}$][**1**]·($C_4H_8O_2$)$_{0.5}$ found % (calc. %): C 40.65 (40.01), H 6.20 (6.25).

Raman (cm^{-1}): 444 (9, δ), 630 (100, ν_s), 1746 (15, ν_{as}).

IR (Nujol mull, cm^{-1}): 1742 (w, ν_{as}).

[Na(18-crown-6)(NC_5H_5)$_2$][**1**]

CHN for [Na(18-crown-6) (NC_5H_5)$_2$][**1**] found % (calc. %): C 39.32 (40.01), H 6.16 (6.20).

Raman (cm^{-1}): 442 (9, δ), 612 (100, ν_s), 1752 (15, ν_{as}).

IR (Nujol mull, cm^{-1}): 1743 (w, ν_{as}).

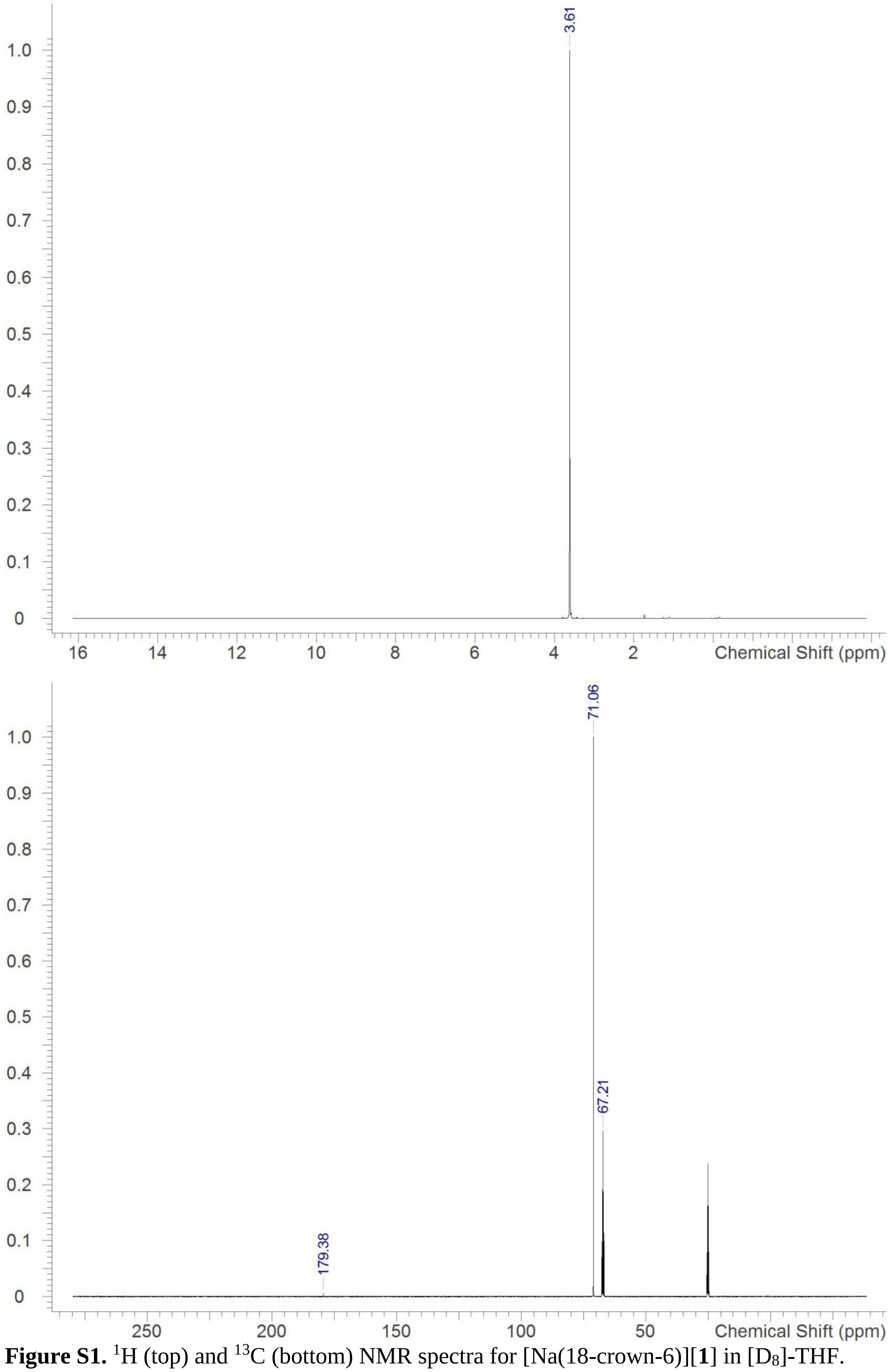

Figure S1. ^{1}H (top) and ^{13}C (bottom) NMR spectra for [Na(18-crown-6)][**1**] in [D_8]-THF.

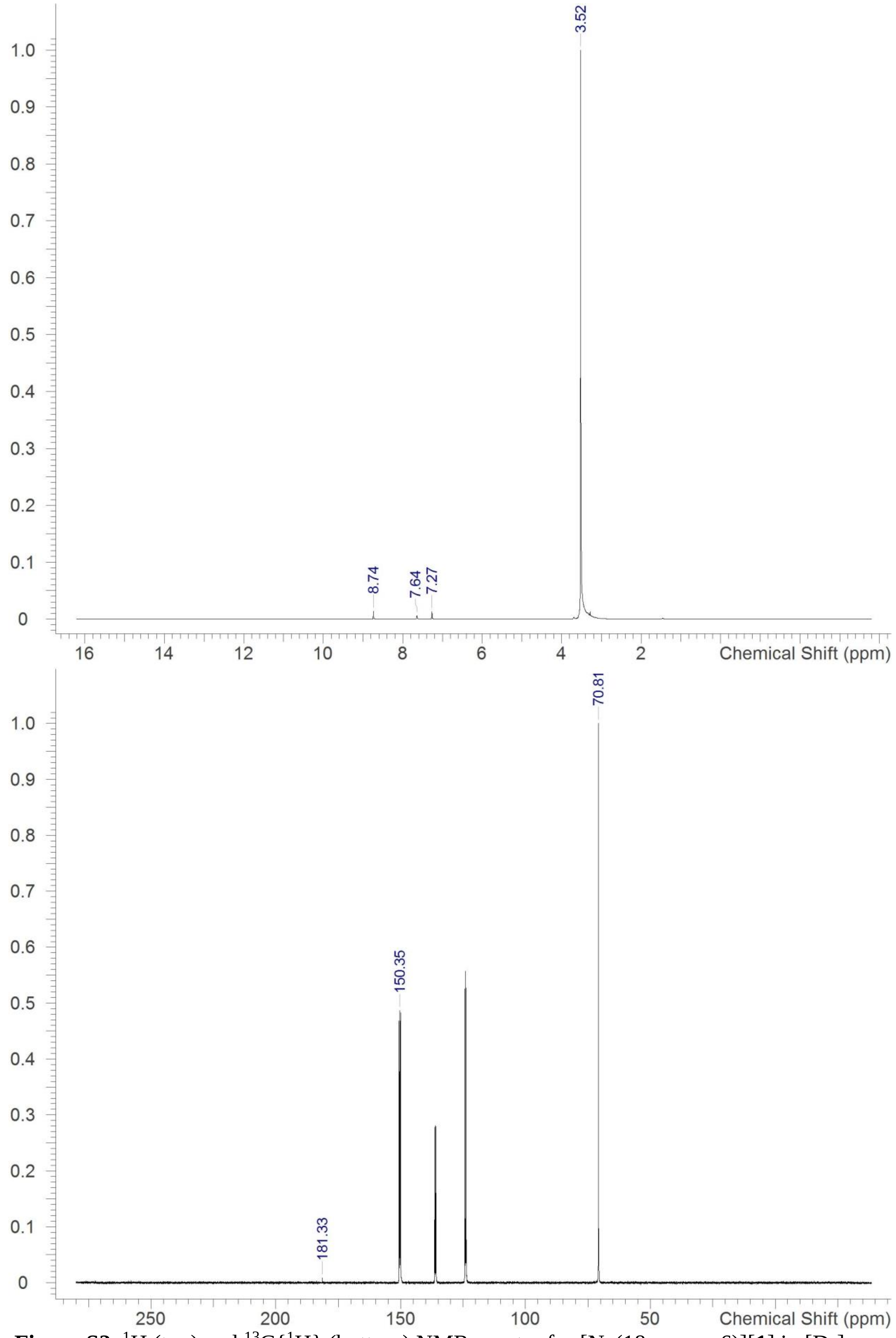

Figure S2. ^{1}H (top) and $^{13}C\{^{1}H\}$ (bottom) NMR spectra for [Na(18-crown-6)][**1**] in [D_5]-py.

1.2 Synthesis of [Na(18-crown-6)][As[C(O)]$_2$CPh$_2$] ([Na(18-crown-6)][2])

[Na(18-crown-6)][AsCO] (39 mg, 0.1 mmol) was dissolved in 0.5 ml [D$_5$]-pyridine. A solution of Ph$_2$CCO (19 mg, 0.1 mmol) in 0.1 ml [D$_5$]-pyridine was added at ambient temperature. The resulting orange solution adopted pale yellow colour and was transferred into an ampoule, layered with *n*-hexane (approx. 10 ml), and left undisturbed for two days, resulting in the formation of colourless yellow crystals above a red oil. The supernatant solution was removed via syringe and the crystals were dried *in vacuo*, affording 8.5 mg of a colourless crystalline solid (0.013 mmol, 13%).

CHN for [Na(18-crown-6)][As[C(O)]$_2$CPh$_2$]·py: found % (calc. %): C 55.31 (55.49), H 5.84 (5.86).

^{1}H NMR ([D$_8$]-THF): 3.53 (s, crown), 7.01 (t, $^3J_{HH}$ = 7.1 Hz, *p*-H), 7.10 (t, $^3J_{HH}$ = 7.2 Hz, *m*-H), 7.56 (d, $^3J_{HH}$ = 7.2 Hz, *o*-H).

^{13}C{^{1}H} NMR ([D$_8$]-THF): 70.7 (s, crown), 106.8 (s, *C*CO), 125.3 (s, *p*-C), 127.5 (s, *o*-C), 128.4 (s, *m*-C), 144.0 (s, *ipso*-C), 230.2 (s, *C*=O).

IR (Nujol mull, cm^{-1}): 696 (vw), 734 (vw), 832 (vw), 900 (vw), 920 (vw), 948 (vw), 959 (vw), 1037 (vw), 1058 (vw), 1105 (vw), 1141 (vw), 1247 (vw), 1298 (vw), 1352 (vw), 1569 (vw), 1600 (vw), 1673 (vw).

Raman (cm^{-1}): 240 (27), 256 (13), 284 (19), 292 (52), 312 (22), 374 (10), 406 (3), 484 (5), 492 (6), 562 (29), 618 (11), 640 (43), 656 (9), 736 (3), 790 (8), 810 (4), 834 (3), 864 (12), 900 (5), 960 (8), 1002 (100), 1030 (22), 1058 (5), 1082 (4), 1156 (16), 1166 (18), 1218 (4), 1246 (5), 1262 (4), 1276 (3), 1316 (3), 1442 (6), 1468 (11), 1566 (11), 1576 (11), 1596 (25), 1674 (33), 1702 (5), 2878 (16), 2908 (23), 2938 (24), 3046 (20), 3064 (20).

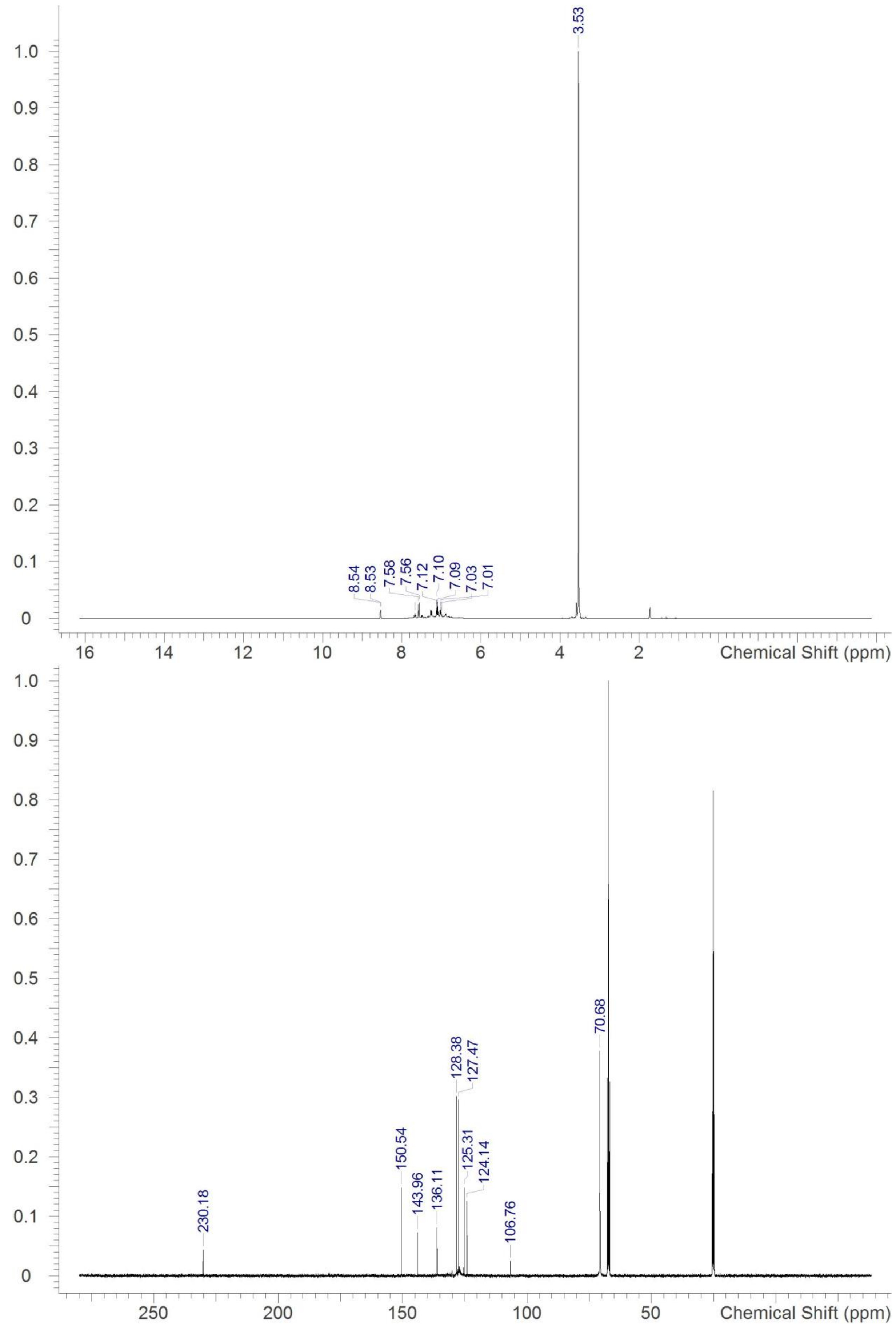

Figure S3. ^1H (top) and ^{13}C (bottom) NMR spectra for [Na(18-crown-6)][**2**] in [D_8]-THF.

1.3 Synthesis of [Na(18-crown-6)][AsC(O)(CNDipp)NDipp] ([Na(18-crown-6)][3])

[Na(18-crown-6)][AsCO] (39 mg, 0.1 mmol) was disolved in 0.5 ml [D_8]-THF. A solution of $C(NDipp)_2$ (36 mg, 0.10 mmol) in 0.1 ml [D_8]-THF was added at ambient temperature. The resulting solution adopted pale yellow colour and was transferred into an ampoule, layered with *n*-hexane (approx. 10 ml) and left undisturbed for a week, resulting in the formation of pale yellow crystals. The supernatant was removed via syringe and the crystals were dried under a dynamic vacuum, affording 54.2 mg of a yellow crystalline solid (0.72 mmol, 72%).

CHN for [Na(18-crown-6)][**3**] found % (calc. %): C 59.35 (60.63), H 7.65 (7.77), N 3.47 (3.72).

^{1}H NMR ([D_8]-THF): 1.18 (d, $^3J_{HH}$ = 6.5 Hz, 12H, CH_3), 1.23 (d, $^3J_{HH}$ = 6.9 Hz, 6H, CH_3), 1.27 (d, $^3J_{HH}$ = 6.9 Hz, 6H, CH_3), 3.49 (s, crown), 3.61 (s, $^3J_{HH}$ = 6.7 Hz, 4H, C*H*CH_3), 6.73 (t, $^3J_{HH}$ = 7.6 Hz, 1H, *p*-C*H*), 6.87 (d, $^3J_{HH}$ = 7.6 Hz, 2H, m-C*H*), 7.05 (d, $^3J_{HH}$ = 7.6 Hz, 2H, m-C*H*), 7.12 (t, $^3J_{HH}$ = 7.3 Hz, 1H, *p*-C*H*).

^{13}C{^{1}H} NMR ([D_8]-THF): 24.1 (s, *C*H_3), 25.3 (s, *C*H_3), 28.0 (s, *C*H_3), 29.7 (s, *C*H), 70.4 (s, crown), 121.4 (s, *p*-C), 122.3 (s, *p*-C), 122.5 (s, *m*-C), 127.3 (s, *m*-C), 136.1 (s, *o*-C), 140.3 (s, *o*-C), 150.3 (s, *i*-C), 150.9 (s, *i*-C), 178.2 (s, N–*C*=N), 195.3 (s, *C*=O).

IR (Nujol mull, cm^{-1}): 728 (vw), 750 (vw), 779 (vw), 806 (vw), 834 (vw), 862 (vw), 937 (vw), 945 (vw), 1105 (w), 1177 (vw), 1198 (vw), 1297 (vw), 1333 (vw), 1352 (vw), 1573 (w), 1598 (w), 1678 (vw), 2725 (vw), 3052 (w).

Raman (cm^{-1}): 242 (39), 266 (45), 306 (51), 340 (53), 442 (60), 518 (45), 582 (98), 618 (48), 780 (24), 806 (22), 848 (43), 884 (44), 952 (23), 1038 (74), 1102 (30), 1240 (60), 1444 (69), 1468 (54), 1584 (89), 1676 (56), 1702 (39), 2862 (81), 2908 (100), 2934 (96), 2956 (78), 3054 (32).

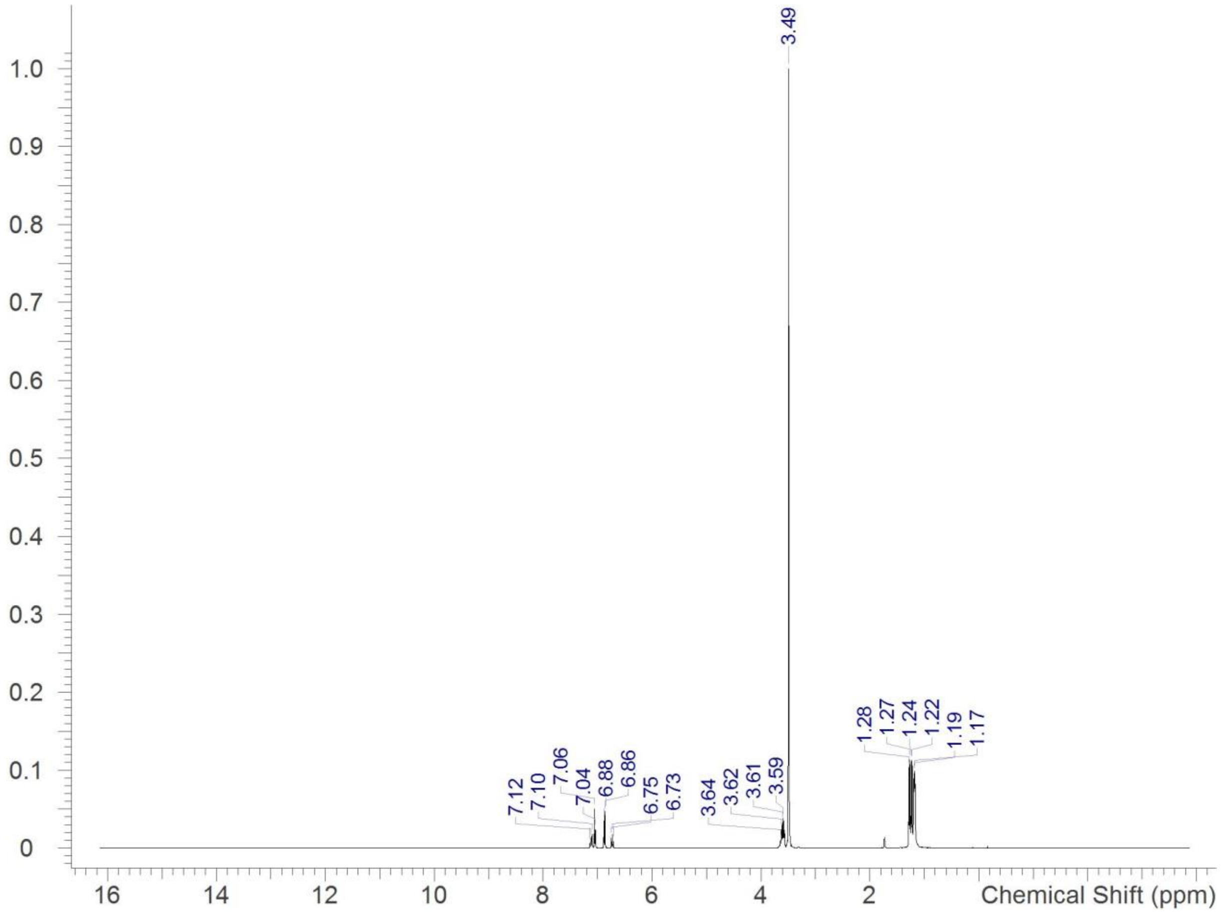

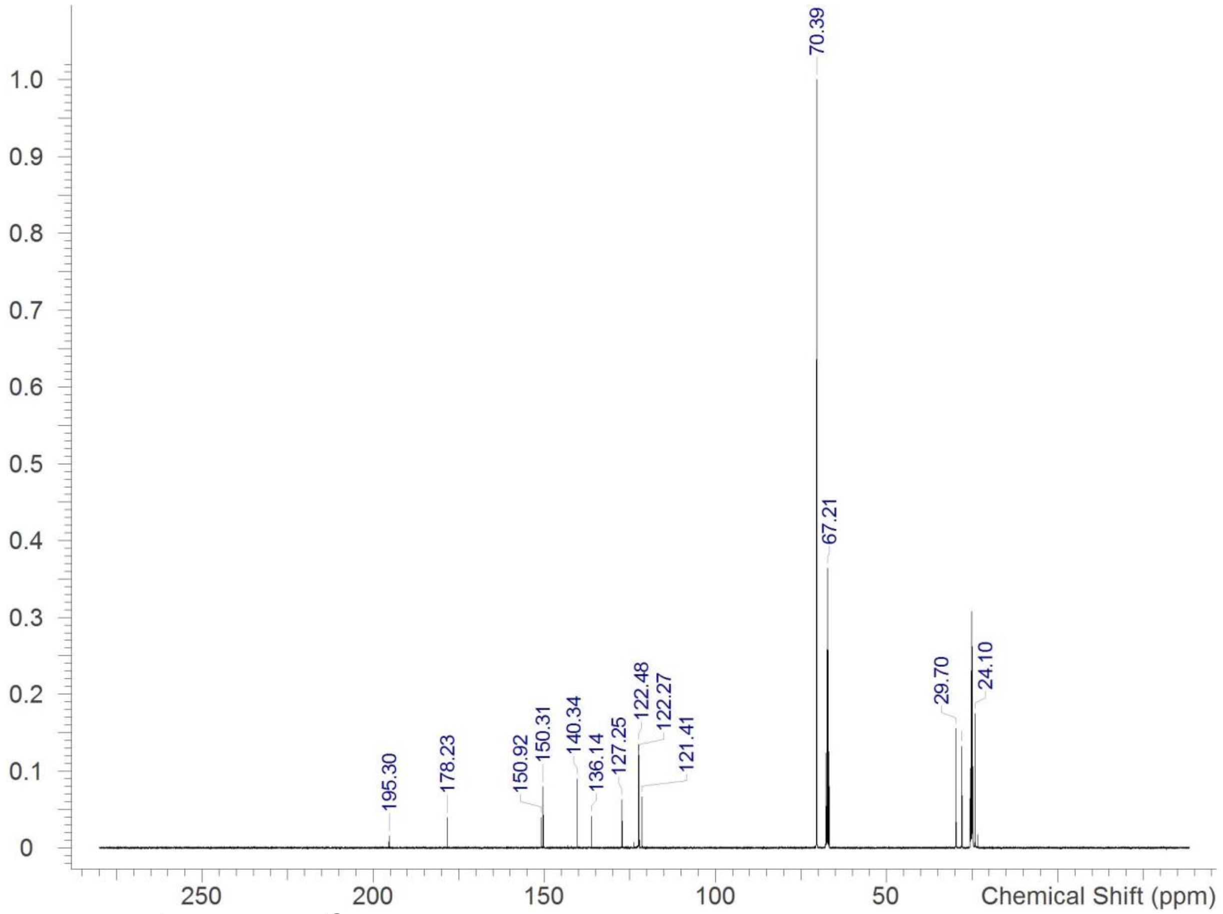

Figure S4. ^{1}H (top) and ^{13}C (bottom) NMR spectra for [Na(18-crown-6)][**3**] in [D_8]-THF.

1.4 Synthesis of [Na(18-crown-6)][As[C(O)]$_2$(NDipp)$_2$] ([Na(18-crown-6)][4]) and [Na(18-crown-6)]$_6$[As$_{10}$][As$_{12}$]

[Na(18-crown-6)][AsCO] (39 mg, 0.1 mmol) was disolved in 0.5 ml [D$_8$]-THF. A solution of C(NDipp)$_2$ (20 mg, 0.1 mmol) in 0.1 ml [D$_8$]-THF was added at ambient temperature. The initially pale yellow solution slowly turned dark red and gas evolution was observable. After standing undisturbed overnight, black crystals deposited. The pale yellowish supernatant was transferred into an ampoule and layered with *n*-hexane. After standing undisturbed overnight, colourless crystals of [Na(18-crown-6)][**4**] were obtained. The supernatant was removed via syringe and the solid was dried under a dynamic vacuum (28.0 mg, 0.036 mmol, 36% As). The red crystals were dried *in vacuo* (6.5 mg, 0.002 mmol, 42% As).

CHN for [Na(18-crown-6)][**4**] found % (calc. %): C – (59.37), H – (7.60), N – (3.64). Satisfactory analyses could repeatedly not be obtained.

^{1}H NMR ([D$_8$]-THF): 1.15 (d, 3H, $^3J_{HH}$ = 6.9 Hz, CH_3), 1.16 (d, 3H, $^3J_{HH}$ = 6.9 Hz, CH_3), 1.21 (d, 3H, $^3J_{HH}$ = 6.9 Hz, CH_3), 1.24 (d, 3H, $^3J_{HH}$ = 6.9 Hz, CH_3), 3.29 (sept, 1H, $^3J_{HH}$ = 6.9 Hz, C*H*CH$_3$), 3.55 (s, crown), 3.62 (sept, 1H, $^3J_{HH}$ = 6.9 Hz, C*H*CH$_3$), A$_2$B (6.99 2H, 7.04 1H, $^3J_{HH}$ = 6.9 Hz, *m*/*p*-C*H*), A$_2$B (7.09 2H, 7.14 1H, $^3J_{HH}$ = 7.5 Hz, *m*/*p*-C*H*).

^{13}C{^{1}H} NMR ([D$_8$]-THF): 23.7 (s, *C*H$_3$), 24.2 (s, *C*H$_3$), 28.7 (s, *C*CH$_3$), 29.4 (s, *C*CH$_3$), 70.6 (s, crown), 122.7 (s, *p*-C*H*), 123.1 (s, *p*-C*H*), 126.0 (s, *C*H), 127.3 (s, *C*H), 136.2 (s), 137.6 (s), 140.0 (s), 148.5 (s), 148.8 (s), 154.6 (s), 179.4 (s, N*C*(N)O), 204.5 (s, As*C*O).

IR (Nujol mull, cm^{-1}): 834 (vw), 844 (vw), 860 (vw), 944 (vw), 1038 (vw), 1105 (vw), 1254 (vw), 1298 (vw), 1538 (vw), 1634 (vw), 1738 (vw).

Raman (cm^{-1}): 298 (46), 326 (49), 376 (48), 444 (37), 514 (19), 538 (19), 596 (44), 630 (70), 738 (19), 860 (31), 888 (33), 956 (20), 1036 (58), 1062 (14), 1104 (23), 1162 (21), 1178 (18),

1232 (19), 1246 (16), 1290 (45), 1346 (18), 1444 (47), 1464 (42), 1540 (17), 1586 (49), 1746 (32), 2866 (86), 2906 (100), 2956 (68), 3060 (33).

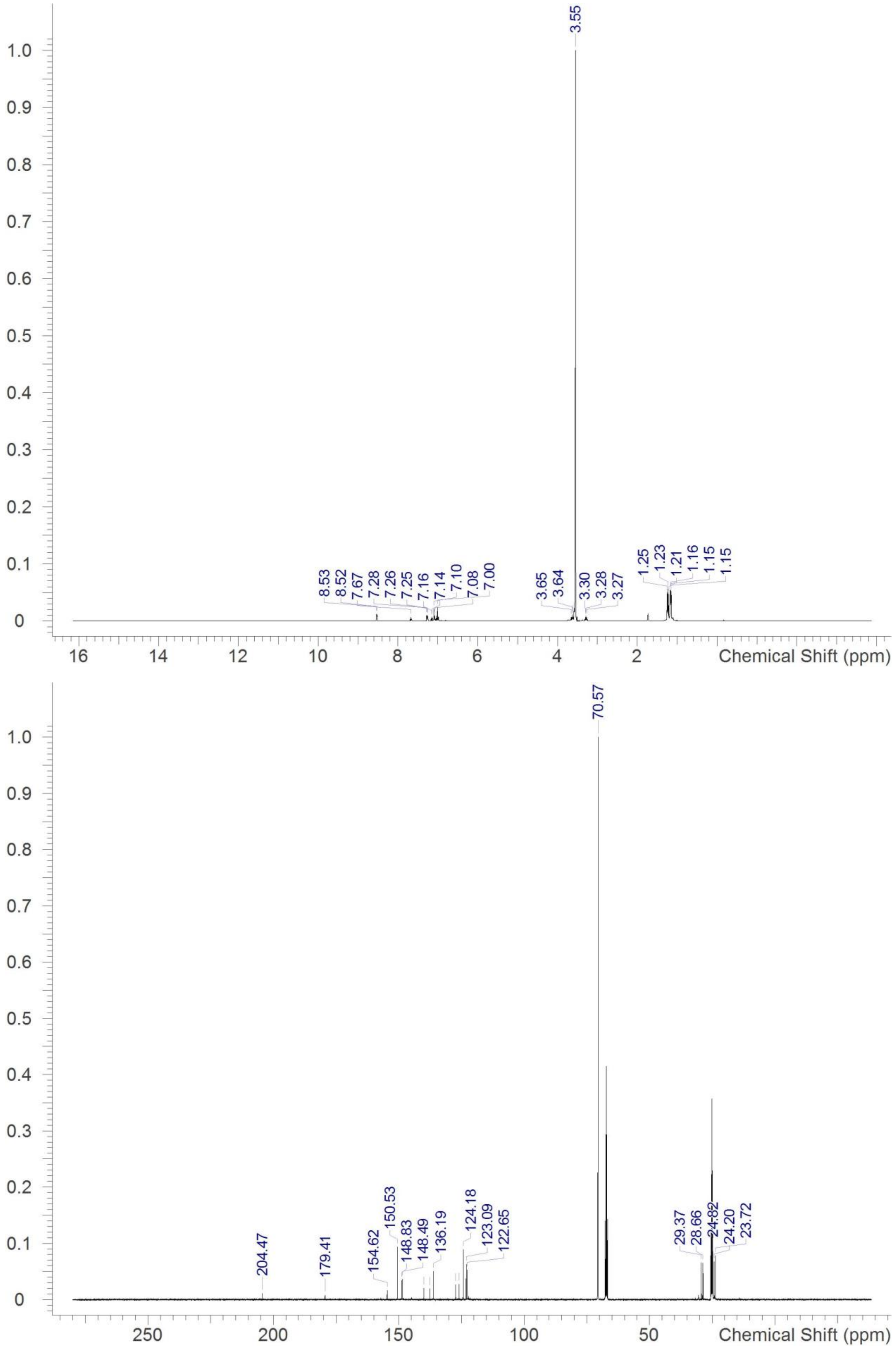

Figure S5. ^{1}H (top) and ^{13}C (bottom) NMR spectra for [Na(18-crown-6)][**4**] in [D_8]-THF.

2. Crystallographic data

Table S1. Selected X-ray data collection and refinement parameters for [Na(18-crown-6)][**1**]·$C_4H_8O_2$, [Na(18-crown-6)$(py)_2$][**1**] and [Na(18-crown-6)$_{1.5}$][**1**].

	[Na(18-crown-6)][**1**]·$C_4H_8O_2$	[Na(18-crown-6)$(py)_2$][**1**]	[Na(18-crown-6)$_{1.5}$][**1**]
Formula	$C_{15}H_{28}AsNaO_8$	$C_{23}H_{34}AsN_2NaO_7$	$C_{19}H_{36}AsNaO_{10}$
CCDC depository number	1457758	1457759	1457760
Fw [g mol^{-1}]	434.28	548.43	522.39
crystal system	$P2_1/c$	$P2_1/c$	$P2_1/c$
space group	monoclinic	monoclinic	monoclinic
a (Å)	10.0034(6)	10.57660(10)	9.0028(2)
b (Å)	17.0910(7)	13.16200(10)	18.4260(3)
c (Å)	12.7834(7)	19.6355(2)	14.6849(2)
β (°)	111.049(7)	92.4930(10)	96.982(2)
V (Å^3)	2039.7(2)	2730.85(4)	2417.95(7)
Z	4	4	4
radiation, λ (Å)	Cu Kα (1.54178)	Cu Kα (1.54178)	Cu Kα (1.54178)
T (K)	150(2)	150(2)	150(2)
ρ_{calc} (g cm^{-3})	1.414	1.334	1.435
μ (mm^{-1})	2.790	2.196	2.515
reflections collected	21120	29149	27442
independent reflections	4237	5681	5044
parameters	383	335	290
R(int)	0.0338	0.0274	0.0248
R1/wR2,[a] $I \geq 2\sigma I$ (%)	4.32/10.75	3.44/8.71	2.80/7.50
R1/wR2,[a] all data (%)	4.66/11.08	4.22/9.32	2.96/7.67
GOF	1.035	1.022	1.034

[a] $R1 = [\Sigma||F_o| - |F_c||]/\Sigma|F_o|$; $wR2 = \{[\Sigma w[(F_o)^2 - (F_c)^2]^2]/[\Sigma w(F_o^2)^2]\}^{1/2}$; $w = [\sigma^2(F_o)^2 + (AP)^2 + BP]^{-1}$, where $P = [(F_o)^2 + 2(F_c)^2]/3$ and the A and B values are 0.0448 and 2.13 for [Na(18-crown-6)][**1**]·$C_4H_8O_2$, 0.0398 and 1.00 for [Na(18-crown-6)$(py)_2$][**1**], and 0.0421 and 0.86 for [Na(18-crown-6)$_{1.5}$][**1**].

Table S2. Selected X-ray data collection and refinement parameters for [Na(18-crown-6)][**2**]·py, [Na(18-crown-6)][**3**] and [Na(18-crown-6)][**4**].

	[Na(18-crown-6)][**2**]·py	[Na(18-crown-6)][**3**]	[Na(18-crown-6)][**4**]
Formula	$C_{32}H_{39}AsNNaO_8$	$C_{38}H_{58}AsN_2NaO_7$	$C_{38}H_{58}AsN_2NaO_8$
CCDC depository number	1457761	1457762	1457763
Fw [g mol^{-1}]	663.55	752.77	768.77
crystal system	*Pn*	$P2_12_12_1$	$P2_1/n$
space group	monoclinic	orthorhombic	monoclinic
a (Å)	14.3797(3)	10.9350(1)	10.4370(1)
b (Å)	10.6505(2)	18.6094(2)	17.7929(3)
c (Å)	20.9181(4)	19.3427(3)	21.5536(3)
α (°)			
β (°)	92.109(2)		100.508(1)
γ (°)			
V (Å^3)	3201.46(11)	3936.12(8)	3935.47(9)
Z	4	4	4
radiation, λ (Å)	Cu Kα (1.54178)	Cu Kα (1.54178)	Cu Kα (1.54178)
T (K)	150(2)	150(2)	150(2)
ρ_{calc} (g cm^{-3})	1.377	1.270	1.298
μ (mm^{-1})	1.991	1.662	1.694
reflections collected	19040	14305	23507
independent reflections	9025	7501	8120
parameters	775	450	516
R(int)	0.0256	0.0219	0.0271
R1/wR2,[a] I ≥ 2σI (%)	3.65/9.90	2.45/5.98	4.22/13.11
R1/wR2,[a] all data (%)	3.75/10.02	2.69/6.25	5.17/14.11
GOF	1.059	1.051	0.805

[a] $R1 = [\Sigma||F_o| - |F_c||]/\Sigma|F_o|$; $wR2 = \{[\Sigma w[(F_o)^2 - (F_c)^2]^2]/[\Sigma w(F_o^2)^2]\}^{1/2}$; $w = [\sigma^2(F_o)^2 + (AP)^2 + BP]^{-1}$, where $P = [(F_o)^2 + 2(F_c)^2]/3$ and the A and B values are 0.0741 and 0.20 for [Na(18-crown-6)][**2**]·py, 0.0270 and 0.59 for [Na(18-crown-6)][**3**], and 0.1239 and 2.70 for [Na(18-crown-6)][**4**].

Table S3. Selected X-ray data collection and refinement parameters for $[Na(18\text{-}crown\text{-}6)]_3[As_{10}]_{0.5}[As_{12}]_{0.5}\cdot 4THF$.

	$[Na(18\text{-}crown\text{-}6)]_3[As_{10}]_{0.5}[As_{12}]_{0.5}\cdot 4THF$
Formula	$C_{52}H_{104}As_{11}Na_3O_{22}$
CCDC depository number	1457764
Fw [g mol^{-1}]	1974.44
crystal system	monoclinic
space group	$P2_1/n$
a (Å)	12.8548(6)
b (Å)	19.6738(7)
c (Å)	15.1460(7)
β (°)	99.508(5)
V (Å^3)	3777.8(3)
Z	2
radiation, λ (Å)	Cu K_α (1.54178)
T (K)	150(2)
ρ_{calc} (g cm^{-3})	1.736
μ (mm^{-1})	6.196
reflections collected	42078
independent reflections	7879
parameters	538
R(int)	0.0368
R1/wR2,[a] I ≥ 2σI (%)	4.03/9.02
R1/wR2,[a] all data (%)	4.66/9.33
GOF	1.086

[a] $R1 = [\Sigma||F_o| - |F_c||]/\Sigma|F_o|$; $wR2 = \{[\Sigma w[(F_o)^2 - (F_c)^2]^2]/[\Sigma w(F_o^2)^2]\}^{1/2}$; $w = [\sigma^2(F_o)^2 + (AP)^2 + BP]^{-1}$, where $P = [(F_o)^2 + 2(F_c)^2]/3$ and the A and B values are 0.0249 and 5.90 for $[Na(18\text{-}crown\text{-}6)]_3[As_{10}]_{0.5}[As_{12}]_{0.5}\cdot 4THF$.

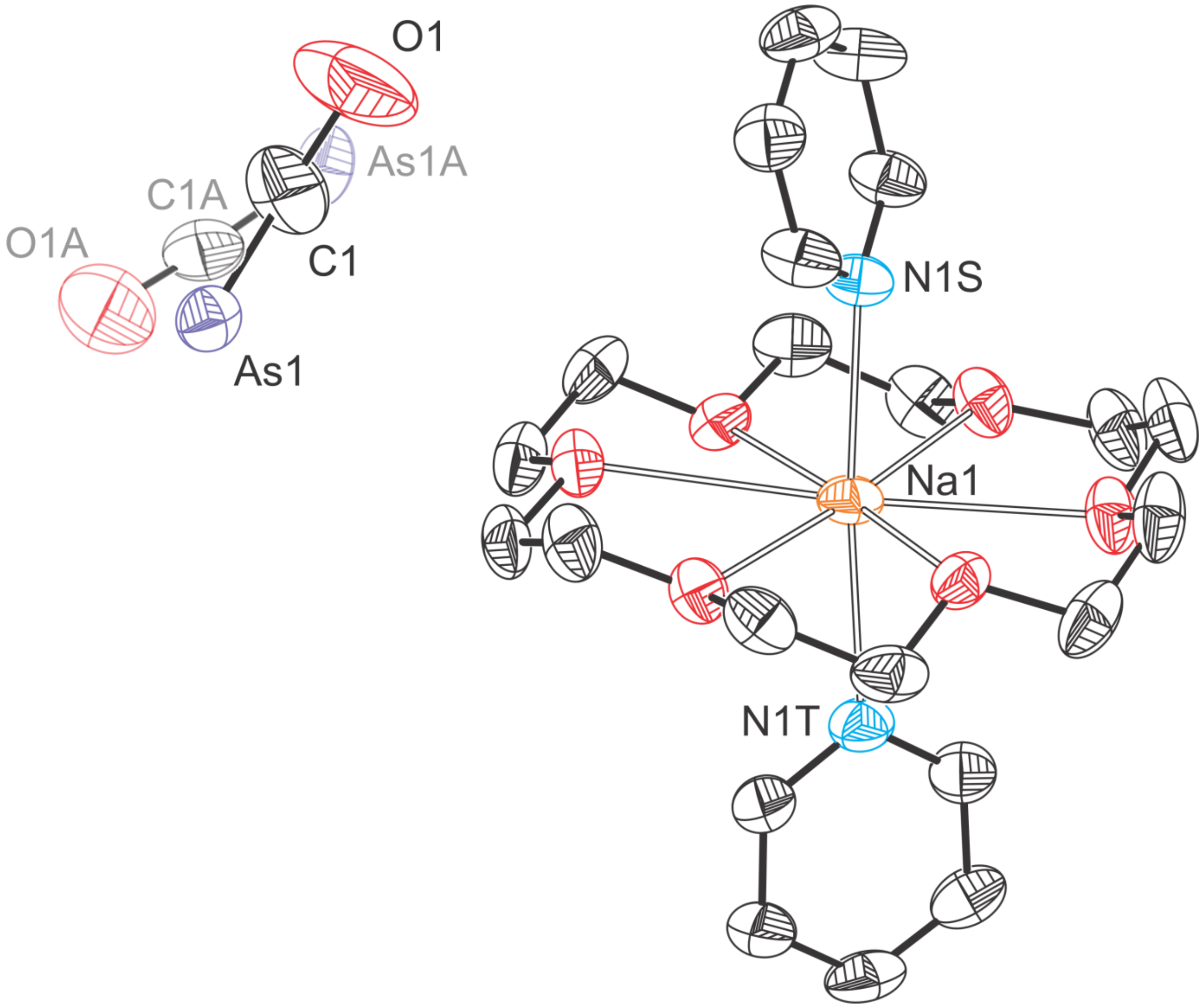

Figure S6. Thermal ellipsoid plot of the atoms in the asymmetric unit of [Na(18-crown-6)(py)$_2$][**1**]. Anisotropic thermal displacement ellipsoids are pictured at the 50% probability level. Hydrogen atoms removed for clarity. The occupation factors of the two disordered $AsCO^-$ components refine as 0.55 and 0.45 (minor component pictured as partially transparent). Selected bond distances (Å) and angles (°): As1–C1, 1.575(5); C1–O1, 1.319(6); As1A–C1A, 1.616(5); C1A–O1A, 1.273(6); As1–C1–O1, 176.8(7); As1A–C1A–O1A, 174.2(8).

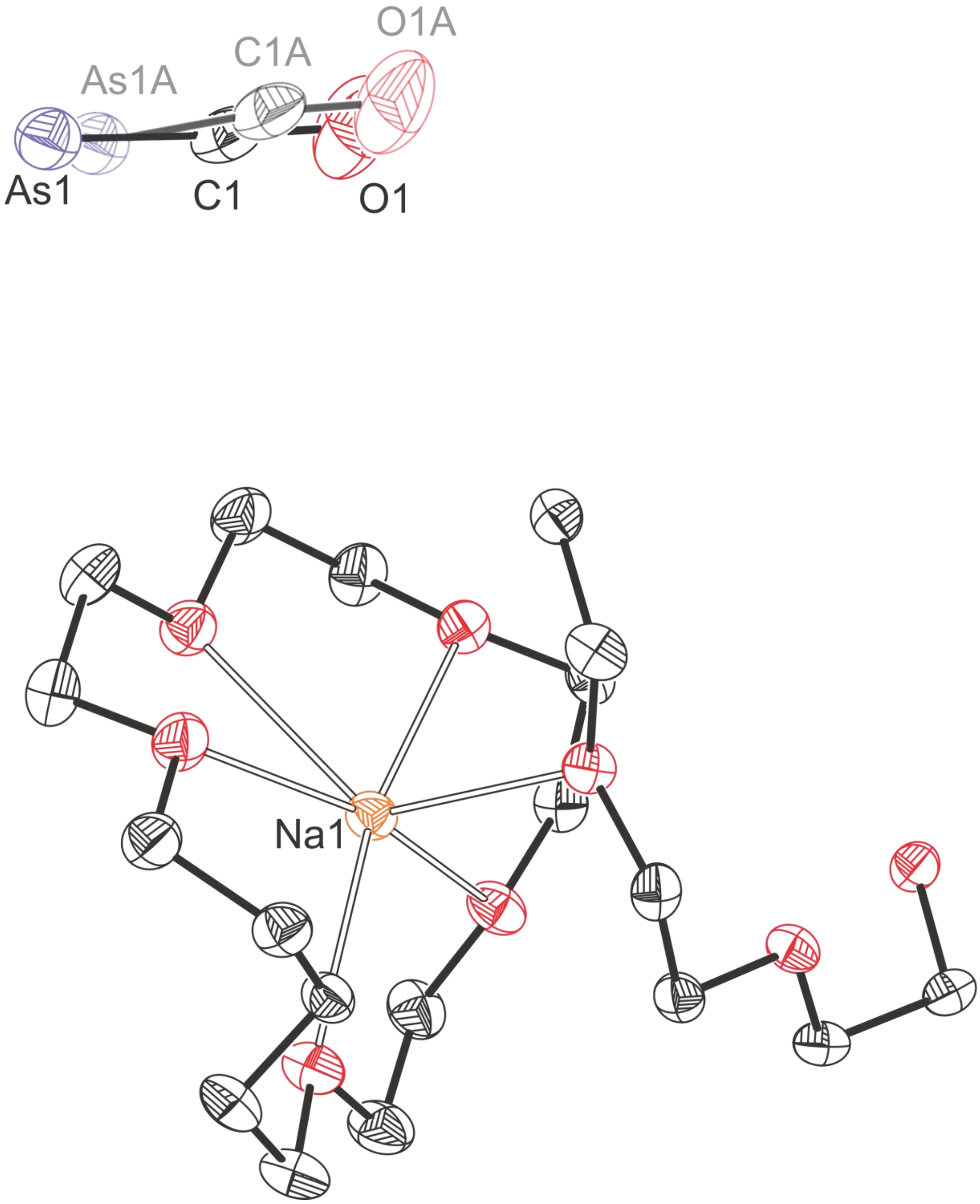

Figure S7. Thermal ellipsoid plot of the atoms in the asymmetric unit of [Na(18-crown-6)$_{1.5}$][**1**]. Anisotropic thermal displacement ellipsoids are pictured at the 50% probability level. Hydrogen atoms removed for clarity. The occupation factors of the two disordered $AsCO^-$ components refine as 0.92 and 0.08 (minor component pictured as partially transparent). Selected bond distances (Å) and angles (°): As1–C1, 1.724(2); C1–O1, 1.194(3); As1A–C1A, 1.662(17); C1A–O1A, 1.185(17); As1–C1–O1, 174.9(3); As1A–C1A–O1A, 173(4).

3. Computational data

Computational details. All computations were performed using Gaussian09[6] utilizing the PBE1PBE level of theory and 6-311G(d,p) basis sets. No solvent corrections were applied. Natural Bond Orbital and Natural Resonance Theory were applied to study the electronic states.[7–9] To investigate aromaticity, Nucleus-Independent Chemical Shifts (NICS) were used.[10]

Selected computed data for $AsCO^{-}$

Computed values (PBE1PBE/6-311G(d,p), gas phase, single ion):

Vibrations (scaled by 0.95, relative Raman activity, relative IR activity):

450 (δ, 19, <1), 601 (ν_{s}, 100, <1), 1801 (ν_{as}, 47, 100).

^{13}C NMR: +173.

Bond metrics: As1–C1: 1.744, C1–O1: 1.188, O1–C1–As1: 180.0°.

Table S4. Computed NICS values for the cyclic compounds.

	NICS(0)	NICS(0.5)	NICS(1)
**2**	+7.8	+2.3	–1.5
**3**	–2.4	–1.4	+0.3
**4**	–5.9	–4.8	–2.4

Table S5. Absolute energies of arsenic cluster anions.

anion	E [a.u.]
D_{2h}-As_{10}^{2-}	–22356.0289953
D_{3}-As_{11}^{3-}	–24591.4814153
D_{4h}-As_{12}^{4-}	–26826.7394112
C_{4h}- As_{12}^{4-}	–26826.7673920

3.1 Optimized Geometries

3.1.1 $AsCO^{-}$

–1 1

C	0.00000000	0.00000000	–1.02207500
As	0.00000000	0.00000000	0.72166600
O	0.00000000	0.00000000	–2.21031500

3.1.2 [Na(18-crown-6)(dioxane)][AsCO]

0 1

Na	0.14644100	0.41235200	0.22936500
O	–1.66090500	1.69496500	0.89391900
O	0.23771600	–1.16448000	2.24348300
O	–0.26697800	1.45174800	–1.99626900
O	1.35869000	1.31824400	2.26144000
As	–4.21994000	0.40743800	0.28528300
C	–2.71737300	1.17445000	0.64450700
C	–0.61083100	0.72296400	–3.15488000
C	0.28622600	2.71675100	–2.26295700
C	–1.60936500	–0.34120900	–2.79783200
O	–1.00153100	–1.30669500	–1.96457500
C	–1.92641400	–2.32137300	–1.62435600
C	–1.22641400	–3.40462800	–0.83737700
O	–0.61271600	–2.96747400	0.35102700
C	–1.51203000	–2.49903000	1.33350900
C	–0.71788400	–2.15866800	2.56204500
C	0.91638600	–0.70064500	3.38535300
C	1.98431700	0.27215400	2.96270900
C	2.17561600	2.44031300	2.03896200
C	1.41418900	3.38441200	1.14947300
O	1.28449900	2.77991600	–0.11882100
C	0.39183500	3.47345900	–0.96767700
O	2.23553700	–0.51293800	–0.46412500
C	3.10887400	0.17311700	–1.34842200
C	2.32531000	–1.92116800	–0.66716200
H	3.00195100	1.23804100	–1.13244000
C	4.53290700	–0.30326100	–1.14975300

H	2.79839100	–0.01706700	–2.38696000
C	3.75307300	–2.37989900	–0.47188000
H	1.99028000	–2.15525800	–1.68735000
H	1.63768800	–2.38673500	0.04063700
O	4.63155700	–1.69700800	–1.34515700
H	4.86998000	–0.03985800	–0.13332900
H	5.20358000	0.16955700	–1.87151300
H	3.85047800	–3.44534900	–0.69266700
H	4.05807900	–2.20935700	0.57362800
H	0.28786500	0.27753600	–3.60773400
H	–1.07401700	1.39185500	–3.89440800
H	–2.47109300	0.11094100	–2.28471100
H	–1.96013300	–0.81520600	–3.72885900
H	–2.33384500	–2.78308000	–2.53888800
H	–2.76799000	–1.88198000	–1.06913400
H	–1.95703900	–4.20178000	–0.62462300
H	–0.42174100	–3.83378900	–1.44099100
H	–2.24537300	–3.27901500	1.59281400
H	–2.07744200	–1.61813400	0.99437000
H	–1.40561400	–1.79180600	3.33684200
H	–0.20007800	–3.05132400	2.94336100
H	1.38515400	–1.53879400	3.92368000
H	0.21095000	–0.20319400	4.06635500
H	2.49444500	0.65093400	3.86099100
H	2.72539600	–0.22248600	2.31843900
H	3.12034000	2.14734200	1.55615100
H	2.41565900	2.93871600	2.99015000
H	0.42185800	3.56883200	1.58170800
H	1.95255400	4.34071700	1.06934900
H	0.76189900	4.49014800	–1.16867300
H	–0.59656900	3.53159100	–0.49465100
H	–0.36302500	3.28102100	–2.94852100
H	1.27708600	2.61898500	–2.73332100

3.1.3 $[As[C(O)]_2CPh_2]^-$ (2)

–1 1

C	0.27666300	1.23538200	–1.13183600
As	0.65933200	2.75713200	–0.00881200
O	0.21118100	1.03529700	–2.33146700
C	0.28943400	1.24371600	1.12954500
C	–1.37102300	–0.38485800	0.00171800
C	–2.02254500	–0.68547800	–1.19836900
C	–2.03537500	–0.64345900	1.20561100
C	–3.29826000	–1.24075400	–1.19417600
C	–3.30820000	–1.20003000	1.20357500
C	–3.94877300	–1.49964800	0.00565800
H	–3.78721600	–1.46000600	–2.13960200
H	–3.80537400	–1.39236800	2.15024100
H	–4.94707800	–1.92740100	0.00946500
O	0.23500500	1.04441700	2.33204400
C	0.03545000	0.19286000	0.00386700
C	1.04690700	–0.94204800	0.00502500
C	1.51161500	–1.48514000	–1.19508700
C	1.52374700	–1.48301000	1.20439500
C	2.42844200	–2.53074500	–1.19712100
H	1.15056800	–1.06884600	–2.12915700
C	2.43603200	–2.53094900	1.19919700
H	1.18618800	–1.05105900	2.14079700
C	2.89682500	–3.06301900	–0.00013500
H	2.77523200	–2.93608500	–2.14442100
H	2.79821000	–2.92961000	2.14356700
H	3.59831700	–3.89298300	–0.00050000
H	–1.52198600	–0.45708400	–2.13467500
H	–1.53993100	–0.40454000	2.13927500
Bq	0.31522000	1.35727000	–0.00181000
Bq	–0.17094200	1.47408300	–0.00089900
Bq	0.80138200	1.24045700	–0.00272100
Bq	–0.65634100	1.59401400	0.00280500
Bq	1.28678100	1.12052600	–0.00642500

3.1.4 [AsC(O)(CNDipp)NDipp]$^{–}$ (3)

–1 1

C	1.49905400	0.05680800	.08843300
As	–0.38118400	0.43176500	2.56669300
O	2.56033400	–0.17121000	2.61702300
C	–0.08512600	0.42239000	0.62466300
N	–0.70311900	0.58664800	–0.47999500
N	1.28942700	0.12881700	0.67931500
C	2.25622600	0.14452500	–0.35632800
C	3.12542500	1.23917300	–0.47892400
C	2.34718700	–0.95593800	–1.22828700
C	4.09755200	1.20717300	–1.48039100
C	3.31485700	–0.93605100	–2.22796600
C	4.19030300	0.13538900	–2.35205000
H	4.78404100	2.04296600	–1.58201500
H	3.39311100	–1.77228400	–2.91479300
H	4.94571400	0.13203600	–3.13282400
C	–2.07038000	0.86279500	–0.50850200
C	–2.48267700	2.16290700	–0.88393500
C	–3.03811900	–0.14134900	–0.28770200
C	–3.83937900	2.43686800	–1.01679300
C	–4.38666500	0.17957300	–0.44048000
C	–4.79626700	1.45486000	–0.79606100
H	–4.15635300	3.43715900	–1.29696200
H	–5.13358600	–0.59251000	–0.27580000
H	–5.85271800	1.68267200	–0.90389400
C	–1.43335200	3.23298400	–1.09512800
H	–0.52420400	2.70290400	–1.40138000
C	–2.63966400	–1.56895100	0.03410100
H	–1.58340900	–1.56120000	0.30999700
C	–1.12645400	3.95002900	0.22355800
H	–2.01360600	4.48573200	0.58202400
H	–0.82766500	3.23931600	0.99987500
H	–0.31821100	4.67935900	0.09330900
C	–1.78968300	4.23638900	–2.18914800
H	–0.93939900	4.89913900	–2.38237100
H	–2.04864600	3.73330600	–3.12574000
H	–2.63437300	4.87338400	–1.90289400
C	–3.39397000	–2.13981800	1.23348300

H	–3.22272300	–1.52132800	2.11806100
H	–4.47289800	–2.20317600	1.05139900
H	–3.03688000	–3.15105100	1.45662300
C	–2.80049600	–2.45757800	–1.20219600
H	–2.21276100	–2.06969500	–2.03898600
H	–2.46679600	–3.48074600	–0.99375000
H	–3.84859100	–2.50159800	–1.52025600
C	3.03207300	2.43375900	0.44808900
H	2.12261700	2.31832000	1.04445900
C	1.44825700	–2.15538000	–1.01648200
H	0.49384300	–1.77150300	–0.64808800
C	4.21808100	2.46568800	1.41505200
H	5.16380800	2.58013100	0.87189600
H	4.12325300	3.30737900	2.10990900
H	4.25358500	1.54159200	1.99563700
C	2.90743200	3.74791300	–0.32406900
H	2.05967900	3.72063000	–1.01415500
H	2.75369200	4.58006300	0.37084200
H	3.81024500	3.96553600	–0.90565100
C	2.04288100	–3.06321300	0.06566900
H	2.98538300	–3.50362000	–0.28059000
H	2.24832500	–2.50410600	0.98133900
H	1.35274600	–3.87882900	0.30743600
C	1.15945400	–2.94237500	–2.29090600
H	0.76734400	–2.29325200	–3.07919200
H	2.05340300	–3.44709400	–2.67488900
H	0.41323400	–3.71686500	–2.08817000
Bq	0.58054200	0.25994100	1.48977300
Bq	0.37663800	–0.71876100	1.46602700
Bq	0.78444700	1.23864400	1.51352000
Bq	0.68249500	0.74929300	1.50164600
Bq	0.47859000	–0.22941000	1.47790000

3.1.5 As_{10}^{2-}

–2 1

As	–0.00001100	1.22558900	1.72848900

As	–1.71661500	1.80248000	–0.00054500
As	–3.25824300	–0.00030600	0.00015900
As	1.71601900	1.80270900	–0.00048200
As	0.00024800	–1.22434200	1.72873800
As	–0.00024800	1.22434200	–1.72873800
As	3.25824300	0.00030600	–0.00015900
As	1.71661500	–1.80248000	0.00054500
As	–1.71601900	–1.80270900	0.00048200
As	0.00001100	–1.22558900	–1.72848900

3.1.6 As_{12}^{4-}

–4 1

As	–1.58684000	–0.82917700	1.97535400
As	–2.81401000	–1.44059600	–0.01009500
As	–1.58940000	–0.79554900	–1.98613300
As	–0.81032100	1.57044600	1.99418300
As	0.81373600	–1.60513700	1.96723100
As	1.44162100	–2.81304500	–0.02460900
As	–0.81373600	1.60513700	–1.96723100
As	1.58940000	0.79554900	1.98613300
As	–1.44162100	2.81304500	0.02461000
As	0.81032100	–1.57044600	–1.99418300
As	1.58684000	0.82917700	–1.97535400
As	2.81401000	1.44059600	0.01009500

3.1.7 As_{12}^{4-}

–4 1

As	–0.55965800	–0.00166900	2.06859800
As	–2.81185700	1.96040900	–0.49314400
As	0.49168500	2.11338700	1.12081200
As	0.49585500	–2.11224700	1.12076100
As	2.80677100	1.96483900	0.49393500
As	–2.80896500	–0.00172800	0.96908600
As	2.80896500	0.00172800	–0.96908600
As	2.81185700	–1.96040900	0.49314400
As	–0.49585500	2.11224700	–1.12076100

As	–2.80677100	–1.96483900	–0.49393500
As	–0.49168500	–2.11338700	–1.12081300
As	0.55965800	0.00166900	–2.06859800

3.1.8 As_{11}^{3-}

–3 1

As	0.00027500	0.00065100	2.06901500
As	0.24633200	–2.24129400	0.99911600
As	–2.06441100	0.90696000	0.99893500
As	1.81862100	1.33429500	0.99846600
As	–0.00007800	–0.00040000	–2.06851300
As	–2.19022800	–0.53676700	–0.99794000
As	0.62983100	2.16532800	–0.99841600
As	1.55971400	–1.62835100	–0.99788100
As	–1.89103000	–2.70460500	–0.00085700
As	3.28798900	–0.28570200	–0.00112100
As	–1.39701400	2.98988500	–0.00080500

3.1.9 $[As[C(O)]_2(NDipp)_2]^-$ (4)

–1 1

C	–0.04912600	0.01988200	–0.49935300
C	–1.06968100	–0.56397300	1.63264300
N	1.06263100	–0.05149100	0.27263000
As	0.77047800	–0.52109600	2.14038500
O	–0.09680400	0.33025900	–1.68564500
O	–2.05650100	–0.77533000	2.32835700
N	–1.19281100	–0.30648600	0.24338500
C	2.34196100	0.24165700	–0.25854600
C	3.24733700	–0.80758700	–0.49252500
C	2.71711000	1.57790000	–0.50845200
C	4.53188800	–0.50278700	–0.94382400
C	4.00741300	1.83422600	–0.96513800
C	4.91630200	0.80646000	–1.17560100
H	5.23433600	–1.31077300	–1.12878400
H	4.30907100	2.85781200	–1.16227700
H	5.91916800	1.02905900	–1.52905400

C	–2.47705800	–0.20407000	–0.36809300
C	–3.10126100	1.05190000	–0.39101400
C	–3.10140400	–1.33320800	–0.91963700
C	–4.35421500	1.17213300	–0.98857200
C	–4.35332300	–1.16296600	–1.51319000
C	–4.97882500	0.07272400	–1.55350500
H	–4.84421100	2.14139900	–1.00955600
H	–4.84211800	–2.03005500	–1.95043300
H	–5.95235300	0.17686300	–2.02418500
C	–2.55456700	–2.75040300	–0.86792000
H	–3.08877500	–3.28381000	–1.66608700
C	–2.45763200	2.26476600	0.24700100
H	–1.46176700	1.97116600	0.58625400
C	1.74857200	2.71589700	–0.25427400
H	0.74635700	2.32574700	–0.44208700
C	2.83764400	–2.25566900	–0.32270400
H	1.84932300	–2.25835700	0.14333100
C	1.81925500	3.16934200	1.20635000
H	1.59134700	2.34024900	1.88297400
H	2.81970300	3.55003500	1.44397000
H	1.09635500	3.97164900	1.39503900
C	1.94003600	3.89633600	–1.20369500
H	1.12411500	4.61384000	–1.07016900
H	2.87618500	4.43411900	–1.01446200
H	1.93609800	3.56898000	–2.24726300
C	3.77320400	–3.02217800	0.61060300
H	4.79493800	–3.07346800	0.21695600
H	3.80735700	–2.54821200	1.59516600
H	3.41662600	–4.04913500	0.74376900
C	2.72298200	–2.93793100	–1.68790200
H	2.02874900	–2.39379100	–2.33336800
H	3.69463000	–2.97816700	–2.19427400
H	2.35624000	–3.96437200	–1.57695700
C	–2.93865100	–3.41839000	0.45772100
H	–2.56854200	–4.44985800	0.48459100
H	–2.52214900	–2.86802300	1.30498300
H	–4.02608700	–3.43921400	0.58220900

C	–1.06257800	–2.91361900	–1.15944600
H	–0.45338400	–2.67561100	–0.28456900
H	–0.85831000	–3.95538700	–1.43113100
H	–0.74114200	–2.26944900	–1.98158600
C	–2.28320900	3.40664800	–0.75392400
H	–1.70070100	3.07116000	–1.61595300
H	–3.24788800	3.77959600	–1.11613600
H	–1.75769700	4.24687300	–0.28698000
C	–3.24123700	2.69603000	1.48835100
H	–3.31050900	1.86448000	2.19397600
H	–2.74157900	3.53392200	1.98674800
H	–4.25708800	3.01458000	1.22671800

4. References

[1] E. C. Taylor, A. McKillop, G. H. Hawks, *Org. Synth.* **1972**, *52*, 36.

[2] J. Cosier, A. M. Glazer, *J. Appl. Cryst.* **1986**, *19*, 105.

[3] CrysAlisPro, Agilent Technologies, Version 1.171.35.8.

[4] a) G. M. Sheldrick, *Acta Cryst.* **2008**, *A64*, 112; b) G. M. Sheldrick, *Acta Crystallogr. Sect. A* **1990**, *46*, 467; c) SHELX2013, *Programs for Crystal Structure Analysis (Release 2013)*, G. M. Sheldrick, University of Göttingen (Germany), **1998**; d) C. B. Hübschle, G. M. Sheldrick, B. Dittrich, *J. Appl. Crystallogr.* **2011**, *44*, 1281–1284.

[5] G. R. Fulmer, A. J. M. Miller, N. H. Sherden, H. E. Gottlieb, A. Nudelman, B. M. Stoltz, J. E. Bercaw, K. I. Goldberg, *Organometallics* **2010**, *29*, 2176–2179.

[6] M. J. Frisch, G. W. Trucks, H. B. Schlegel, G. E. Scuseria, M. A. Robb, J. R. Cheeseman, G. Scalmani, V. Barone, B. Mennucci, G. A. Petersson, H. Nakatsuji, M. Caricato, X. Li, H. P. Hratchian, A. F. Izmaylov, J. Bloino, G. Zheng, J. L. Sonnenberg, M. Hada, M. Ehara, K. Toyota, R. Fukuda, J. Hasegawa, M. Ishida, T. Nakajima, Y. Honda, O. Kitao, H. Nakai, T. Vreven, J. A. Montgomery, J. E. Peralta, F. Ogliaro, M. Bearpark, J. J. Heyd, E. Brothers, K. N. Kudin, V. N. Staroverov, R. Kobayashi, J. Normand, K. Raghavachari, A. Rendell, J. C. Burant, S. S. Iyengar, J. Tomasi, M. Cossi, N. Rega, J. M. Millam, M. Klene, J. E. Knox, J. B. Cross, V. Bakken, C. Adamo, J. Jaramillo, R. Gomperts, R. E. Stratmann, O. Yazyev, A. J. Austin, R. Cammi, C. Pomelli, J. W. Ochterski, R. L. Martin, K. Morokuma, V. G. Zakrzewski, G. A. Voth, P. Salvador, J. J. Dannenberg, S. Dapprich, A. D. Daniels, Ö. Farkas, J. B. Foresman, J. V. Ortiz, J. Cioslowski, D. J. Fox, *Gaussian 09 Rev. A.02*,

Gaussian Inc., Wallingford CT, **2009**.

[7] E. D. Glendening, C. R. Landis, F. Weinhold, *J. Comput. Chem.* **2013**, *34*, 1429–1437.

[8] E. D. Glendening, F. Weinhold, *J. Comput. Chem.* **1998**, *19*, 593–609.

[9] E. D. Glendening, F. Weinhold, *J. Comput. Chem.* **1998**, *19*, 610–627.

[10] P. von R. Schleyer, C. Maerker, A. Dransfeld, H. Jiao, N. J. R. van E. Hommes, *J. Am. Chem. Soc.* **1996**, *118*, 6317–6318.
